# Perioperative administration of sub-anesthetic ketamine/esketamine for preventing postpartum depression symptoms: A trial sequential meta-analysis

**DOI:** 10.1371/journal.pone.0310751

**Published:** 2024-11-18

**Authors:** Kuo-Chuan Hung, Chia-Li Kao, Yi-Chen Lai, Jen-Yin Chen, Chien-Hung Lin, Ching-Chung Ko, Chien-Ming Lin, I-Wen Chen

**Affiliations:** 1 Department of Anesthesiology, Chi Mei Medical Center, Tainan City, Taiwan; 2 Department of Anesthesiology, E-Da Hospital, I-Shou University, Kaohsiung, Taiwan; 3 School of Medicine, College of Medicine, National Sun Yat-sen University, Kaohsiung, Taiwan; 4 Department of Medical Imaging, Chi Mei Medical Center, Tainan City, Taiwan; 5 Department of Health and Nutrition, Chia Nan University of Pharmacy and Science, Tainan City, Taiwan; NYU Grossman School of Medicine: New York University School of Medicine, UNITED STATES OF AMERICA

## Abstract

**Objective:**

Postpartum depression (PPD) is a major mental health issue affecting 10%–15% of women globally. This meta-analysis synthesized updated evidence on sub-anesthetic ketamine/esketamine’s efficacy in preventing PPD.

**Methods:**

Randomized controlled trials (RCTs) comparing ketamine/esketamine to a placebo for PPD prevention were searched without language restriction. Primary outcomes were PPD risk at 1- and 4–6-week postpartum. Secondary outcomes included the difference in depression scores and risk of adverse events. Trial sequential analysis (TSA) was conducted to validate the reliability.

**Results:**

A meta-analysis of 22 RCTs (n = 3,463) showed that ketamine/esketamine significantly decreased PPD risk at 1- (risk ratio [RR], 0.41; 95% confidence interval [CI], 0.3–0.57) and 4–6-week (RR, 0.47; 95%CI, 0.35–0.63) follow-ups. Consistently, participants receiving ketamine/esketamine had lower depression-related scores at 1- (standardized mean difference [SMD], −0.94; 95%CI, −1.26 to −0.62) and 4–6-week (SMD, −0.89; 95%CI, −1.25 to −0.53) follow-ups. Despite potential publication bias, TSA confirmed the evidence’s reliability. Subgroup analysis showed that ketamine/esketamine’s preventive effect on 1-week PPD was consistent, regardless of administration timing, type of agents, or total dosage (<0.5 vs. ≥0.5 mg/kg). For the 4–6-week period, PPD risk was favorably reduced only with postoperative administration or the use of esketamine, with the total dosage having no observed influence. Participants on ketamine/esketamine experienced more frequency of hallucinations (RR, 4.77; 95%CI, 1.39–16.44) and dizziness (RR, 1.36; 95%CI, 1.02–1.81).

**Conclusion:**

Our findings advocate for the postoperative administration of low-dose ketamine/esketamine to avert PPD, which needed additional research for confirmation.

## 1. Introduction

Postpartum depression (PPD) represents a significant mental health concern, affecting approximately 10%–15% of women globally [1, 2]. In low- and middle-income countries, the PPD prevalence ranges from 21.9% to 33.82% [3, 4]. On the other hand, high-income countries exhibit a notably lower incidence, with estimates between 10–15% [5]. This disparity underscores the widespread nature of PPD, with a notably higher occurrence in low-income and middle-income countries. Symptoms of PPD typically manifest within 4–6 weeks following childbirth and include persistent low mood, diminished interest in usual activities, fatigue, feelings of worthlessness or guilt, impaired concentration, and suicidal thoughts [6, 7]. PPD can significantly impact the maternal quality of life and lead to long-term emotional, functional, and economic burdens on women, families, and society [6]. Furthermore, the impacts of PPD are widespread and not solely limited to the mothers who experience it. Children born to mothers with PPD frequently face a higher likelihood of being underweight and encountering developmental challenges [8–10]. Additionally, PPD increases the risk of poor mother–baby attachment and, in the longer term, impairs offspring’s emotional, social, and cognitive development [9, 11]. Evidence on the efficacy and safety of antidepressants (e.g., selective serotonin reuptake inhibitors) for preventing or treating PPD remains lacking [12, 13]. Consequently, investigating alternative prophylactic strategies for preventing PPD onset is urgently needed.

Ketamine and its S-enantiomer, esketamine, have exhibited rapid and enduring antidepressant properties, particularly in individuals afflicted by major depressive disorder and treatment-resistant depression [14, 15]. Their antidepressant effects are believed to involve increased glutamate transmission, upregulation of α-amino-3-hydroxy-5-methyl-4-isoxazolepropionic acid (AMPA) receptors, enhanced brain-derived neurotrophic factor signaling, and synaptic dysfunction reversal in mood-regulating brain regions [16]. In addition to the therapeutic effects of ketamine/esketamine on depression, several clinical studies have suggested that perioperative intravenous administration of sub-anesthetic doses of ketamine or esketamine decreases PPD incidence [17–19], indicating a potential prophylactic benefit. Consistently, a recent meta-analysis on 2,087 cases reported that patients treated with perioperative ketamine had a lower incidence of PPD in the first postpartum week than those receiving placebo [20]. Despite this encouraging finding, the preventive effect of ketamine for PPD at 4–6-week postpartum was not investigated in that meta-analysis [20]. Accordingly, whether such prophylactic intervention can truly reduce the incidence of PPD, considering its typical onset within 4–6-week postpartum, remains unclear. Recent studies have increasingly focused on the efficacy of esketamine in treating treatment-resistant depression [19, 21–25]. To provide up-to-date insights, there is a pressing need to review and consolidate evidence concerning the prophylactic efficacy of esketamine in reducing PPD incidence.

To bridge this knowledge gap, our systematic review and meta-analysis aimed to synthesize current evidence on the effectiveness of ketamine/esketamine in preventing PPD, with a particular emphasis on the 4–6-week postpartum period. The primary outcomes of interest are the association between ketamine/esketamine use and occurrence of PPD at 1 and 4–6 weeks, whereas secondary outcomes are to investigate the difference in depression-associated scores and risk of adverse events with ketamine/esketamine use.

## 2. Method

This meta-analysis was prospectively registered with PROSPERO. This study adhered to the Preferred Reporting Items for Systematic Reviews and Meta-Analyses Statement (PRISMA) [26] (S1 Table) and AMSTAR (Assessing the methodological quality of systematic reviews) [27] guidelines when reporting its findings.

### 2.1 Data sources and literature search

We performed a comprehensive literature search in the following databases from inception to October, 2023: Medline, Embase, Cochrane Central Register of Controlled Trials, and Google Scholar. The search strategy combined terms related to the population (e.g., cesarean section [CS] or post-CS), interventions (e.g., “ketamine” and “esketamine”), outcomes (e.g., “postpartum depression” and “postnatal depression”), and study design (e.g., “randomized controlled trials [RCTs]”). To facilitate literature search, medical subject headings and text words were used. Search strategies were developed with guidance from a medical librarian. The search strategy for one of the databases is summarized in S2 Table. No language or date restrictions were imposed. Additionally, we searched databases of Chinese scholarly and technical literature, including the China National Knowledge Infrastructure (CNKI), CQVIP Database for Chinese Technical Periodicals, Chinese Biomedical Literature, and Wanfang Database. Reference lists of all included trials and relevant systematic reviews were also manually screened. Authors of included studies were contacted for missing information if required.

### 2.2. Inclusion and exclusion criteria

We included RCTs investigating the efficacy and safety of perioperative use of sub-anesthetic ketamine/esketamine in preventing PPD. We used the following population–intervention–comparators–outcomes criteria to screen eligible articles: Population: parturients without a history of depressive disorder undergoing CS under regional anesthesia or general anesthesia; Intervention: intraoperative or postoperative use of ketamine/esketamine via the intravenous route at sub-anesthetic doses for preventing PPD; Comparators: placebo control (e.g., equal volume of normal saline) or standard care; Outcome: the incidence of PPD at 1- and 4–6-week postpartum.

We excluded non-randomized trials, non-peer-reviewed articles, observational studies, case reports/series, and ongoing trials with unavailable data. Studies involving patients with mixed psychiatric disorders were excluded unless PPD-specific data can be extracted. Trials using ketamine or esketamine primarily for anesthesia or procedural sedation were also excluded. Any disagreements during study selection were resolved through discussion or consultation with a third team member.

### 2.3. Outcomes and definition

Considering that all studies included employed screening tools to identify the occurrence of PPD, we opted to use the term “PPD symptoms” (PPDS) instead of “PPD” in current meta-analysis from this point forward. This choice more accurately reflects the fact that the occurrence of PPD have not been formally diagnosed. The primary outcomes were incidence of positive PPD screening (i.e., the presence of PPDS) at 1 week and 4–6 weeks postpartum. PPDS occurrence was defined on the basis of individual study. Secondary outcomes included the difference in depression-associated scores measured using validated clinician-administered scales or self-report scales (e.g., Edinburgh Postnatal Depression Scale [EPDS]) at 1 or 4–6-week postpartum as well as adverse events (e.g., postoperative nausea and vomiting [PONV]) related to ketamine/esketamine use.

### 2.4. Data extraction and quality of assessment

Two reviewers independently extracted data from included studies using a pre-tested data extraction form. The following were the extracted information: study characteristics (e.g., author, year, country, and sample size); participant characteristics (e.g., age); intervention details (e.g., drug, dosage, route, and time of administration [i.e., intraoperative or postoperative period]); outcomes (e.g., definition and assessment tools); and other parameters, including the follow-up duration. Depression rating scale scores reported as either continuous or dichotomized outcomes were included. Study authors were contacted for any missing, unclear, or additional data required. Two reviewers crosschecked the extracted data to ensure accuracy.

Included RCTs was assessed for quality using the RoB 2.0 [28], which evaluates bias across the following five domains: bias arising from the randomization process, bias due to deviations from the intended interventions, bias due to missing outcome data, bias in outcome measurement, and bias in the selection of the reported results. Two reviewers independently assessed the study quality, with any disagreements resolved through consensus.

### 2.5. Certainty of evidence assessment

We assessed the certainty of evidence for each outcome using the Grading of Recommendations, Assessment, Development, and Evaluation (GRADE) approach [29]. This method considers domains such as the risk of bias, inconsistency, indirectness, imprecision, and publication bias to determine the overall confidence in the effect estimates. The certainty of evidence was upgraded if certain criteria were met, such as the presence of a large effect size [30]. Two reviewers independently evaluated the certainty of the evidence, rating it as high, moderate, low, or very low. Discrepancies were resolved through discussion or consultation with a third reviewer.

### 2.6. Data synthesis

Meta-analyses were conducted using Cochrane Review Manager (RevMan 5.3; Copenhagen: The Nordic Cochrane Centre, The Cochrane Collaboration, 2014). For continuous outcomes, we calculated standardized mean differences (SMDs) with 95% confidence intervals (CIs). The interpretation of the SMD effect size is as follows: a value below 0.2 is indicative of a small effect, a value around 0.5 suggests a moderate effect, and a value of 0.8 or above denotes a large effect [31]. Dichotomous data were expressed as risk ratios (RRs) with 95% CIs. We assessed heterogeneity using the I^2^ statistic, considering an I^2^ value of >50% as indicative of substantial heterogeneity. Anticipating significant heterogeneity, we employed a random-effects model for the analysis. We investigated the sources of heterogeneity through pre-specified subgroup analyses on the basis of the drug used (i.e., ketamine vs. esketamine), dosing regimen, and timing of intervention (i.e., intraoperative vs. postoperative periods). To evaluate the impact of total dosage on treatment response, we planned to perform univariate meta-regression. To examine the robustness of the results, we performed a leave-one-out sensitivity analysis. We evaluated publication bias both visually, using funnel plots when at least ten studies were available, and quantitatively, with Egger’s regression test. A p-value of <0.05 was set as the criterion for statistical significance in this meta-analysis.

We adjusted the participant numbers to avoid overcounting in studies with multiple intervention arms. If a study had two intervention groups and one control group, the control group’s participant count was split equally for comparison with each intervention group. For continuous outcomes, adjusting means and standard deviations was not required. However, for dichotomous outcomes, the number of participants who experienced events was divided accordingly. For example, in a study with 55 events among 75 participants in the control group, we would split the data into two datasets: one with 28 events in 38 participants and another with 27 events in 37 participants. Thus, a single study may contribute to multiple datasets for comparison.

Considering the possibility of false-positive results due to multiple testing, we conducted a trial sequential analysis (TSA) using TSA viewer version 0.9.5.10 Beta to evaluate the strength of the cumulative evidence regarding the primary outcome. This involved assessing the interaction between the TSA boundary and the cumulative Z-curve following the calculation of the trial sequential monitoring and the required information size thresholds. If the cumulative Z-curve crosses the TSA boundary, it signifies a substantial level of evidence supporting the expected intervention effect, negating the necessity for additional studies. Conversely, if no intersection is noted between the cumulative Z-curve and the TSA boundary, it indicates that the evidence is insufficient to form a definitive conclusion.

## 3. Results

### 3.1 Study selection

**Fig 1:**
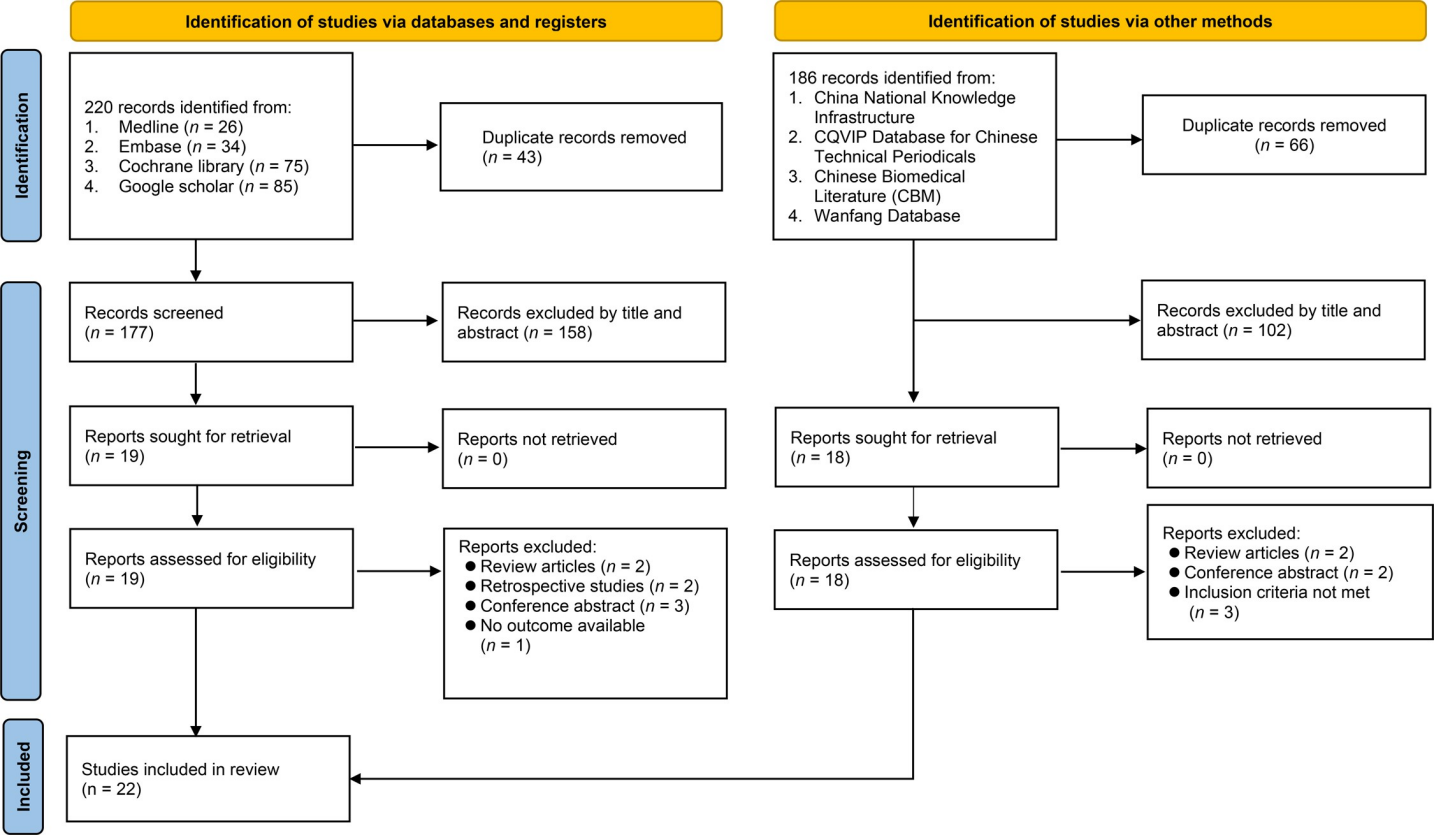
An overview of the selection process for studies.

The study selection process for the current meta-analysis is depicted in Fig 1. The initial search yielded a total of 220 records from four databases. Following the removal of 43 duplicate records, the titles and abstracts of the remaining studies were screened, thereby leading to the exclusion of additional 158 records. Nineteen records were subsequently assessed in detail for eligibility. Of these, the following were excluded for various reasons: review articles (n = 2), retrospective studies (n = 2), conference abstracts (n = 3), and one study for which no outcomes were available (S3 Table). Ultimately, 11 studies were identified from these databases. From the Chinese databases (e.g., CNKI), 186 records were identified, which were reduced to 120 after removing 66 duplicates. Further screening excluded 102 records, and of the 18 retrieved for full-text assessment, seven were further excluded (two review articles, two conference abstracts, and three not meeting the inclusion criteria), resulting in 11 studies being included in our review. A total of 22 RCTs published between 2013 and 2023 were included in the current meta-analysis [17–19, 32–50].

### 3.2. Characteristics and quality of studies

Table 1 summarizes the characteristics of 22 studies, which involved a total of 3,463 women who underwent CS under general anesthesia (n = 1) [48] or regional spinal anesthesia (n = 21) [17–19, 32–36, 38–47, 49, 50]. The mean age of women across 20 RCTs ranged from 26–33 years, whereas two RCTs [17, 49] did not report detailed information. Two types of ketamine including racemic ketamine (11 trials) [17, 32, 33, 35, 39–42, 44, 47, 48] and esketamine (11 trials) [18, 19, 34, 36, 38, 43, 45, 46, 49–51] were used for PPDS prevention. Ketamine/esketamine administration occurred during the following two potential periods: intraoperative (i.e., intravenous bolus or continuous infusion) and/or postoperative. Of the 11 studies that administered ketamine/esketamine postoperatively, 10 used patient-controlled intravenous analgesia (PCIA) initiated immediately following surgery to deliver the agents [17, 19, 36, 42, 43, 45–47, 50, 51]. The remaining study [38] administered esketamine intravenously for 10 min immediately following surgery. Intraoperative ketamine/esketamine dosages ranged from 0.2–0.5 mg/kg, whereas postoperative usage varied from 0.1–2 mg/kg.

**Table 1:** Characteristics of studies (*n* = 22).

Studies	Age (years)	Type of Anesthesia	*n* (I)	*n* (C)	Timing	Type of ketamine	Route and dosage (mg/kg)	PPDS definition	Follow-up time	Country
Alipoor 2021	27/28	GA	67	67	a	ketamine	e (0.5)	EPDS≥13	2w, 4w	Iran
Ge 2019	28/28/28	SA	120/120	120	b	ketamine	PCIA (0.2/0.8)	EPDS⁋	1w, 6w	China
Han 2022	32/32	SA	122	153	b	esketamine	PCIA (0.5)	EPDS≥10	3d, 2w, 4w	China
Liu 2023	30/30	SA	62	61	a+b	esketamine	e (0.25) + PCIA (1.25)	EPDS≥13	3d, 6w, 90d, 180 d	China
Liu 2013	29/28	SA+EA	40	40	a	ketamine	e (0.5)	EPDS≥10	1d, 3 d	China
Liu 2021	28/29	SA	59	58	a+b	esketamine	e (0.5) + PCIA (0.5)	EPDS≥13	3d, 6w	China
Luo 2019	27/28	SA+EA	40	40	b	ketamine	PCIA (2)	EPDS≥13	3d	China
Lv 2015	30/29	SA+EA	47	47	a	ketamine	e (0.5)	EPDS≥10	3d	China
Ma 2019	82%/80%†	SA	327	327	a+b	ketamine	e (0.5) + PCIA (160 mg)	EPDS >9	4d, 6w	China
Monks 2022	30/33	SA	8	7	a	ketamine	e (0.5)	EPDS>12	1d, 2d, 3w, 6w	USA
Shen 2022	29/30	SA+EA	102	100	a	esketamine	e (0.25)	EPDS ≥9	1w, 2w, 4w	China
Shi 2020	30/30/30	SA	67/62	70	a	ketamine	e (0.3/0.5)	EPDS≥13	1d, 3d,1w, 6w	China
Sun 2023	20–41§	SA+EA	135/135	141	a	esketamine	e (0.2/0.4)	EPDS≥13	1d, 3d, 1w, 6w	China
Wang 2023a	28/27/28/28	SA+EA	28/30/29	29	b	esketamine	PCIA (0.1/0.2/0.4)	EPDS≥13	1w, 6w	China
Wang 2023b	28/28	SA+EA	58	57	a	esketamine	e (0.2)	EPDS >9	1w, 6w	China
Wang 2022	29/28/28/29	SA+EA	38/40/39/	39	b	esketamine	PCIA (0.1/0.2/0.4)	EPDS⁋	1w, 6w	China
Wu 2023	28/28	SA+EA	120	120	b	esketamine	f (1)	EPDS≥10	3d, 1w, 6w	China
Xu 2017	31/32	SA	162	163	a	ketamine	e (0.25)	EPDS≥10	3d, 6w	China
Yang 2023a	26/28	SA+EA	40	40	b	esketamine	PCIA (0.4)	EPDS≥9	3d, 6w	China
Yang 2023b	32/32/32	SA	99/99	97	a+b	esketamine	e (0.25) + PCIA (1/2)	EPDS >9	1w, 6w	China
Yao 2020	30/30	SA	153	155	a	ketamine	e (0.25)	EPDS ≥9	1d, 2w, 4w	China
Zhang 2016	28/28	SA+EA	30	30	a	ketamine	e (0.5)	PDSS>75	1d, 4d	China

I: intervention group; C: control group; a: intraoperative period; b: postoperative period; e: iv bolus or infusion; PCIA: intravenous patient-controlled analgesia; f: esketamine administered intravenously for 10 min immediately following surgery

⁋no mention; †(<35years); §range; d: days; w: weeks; SA: spinal anesthesia; EA: epidural anesthesia; PPDS: postpartum depression symptoms. PDSS: Postpartum Depression Screening Scale.

**Fig 2:**
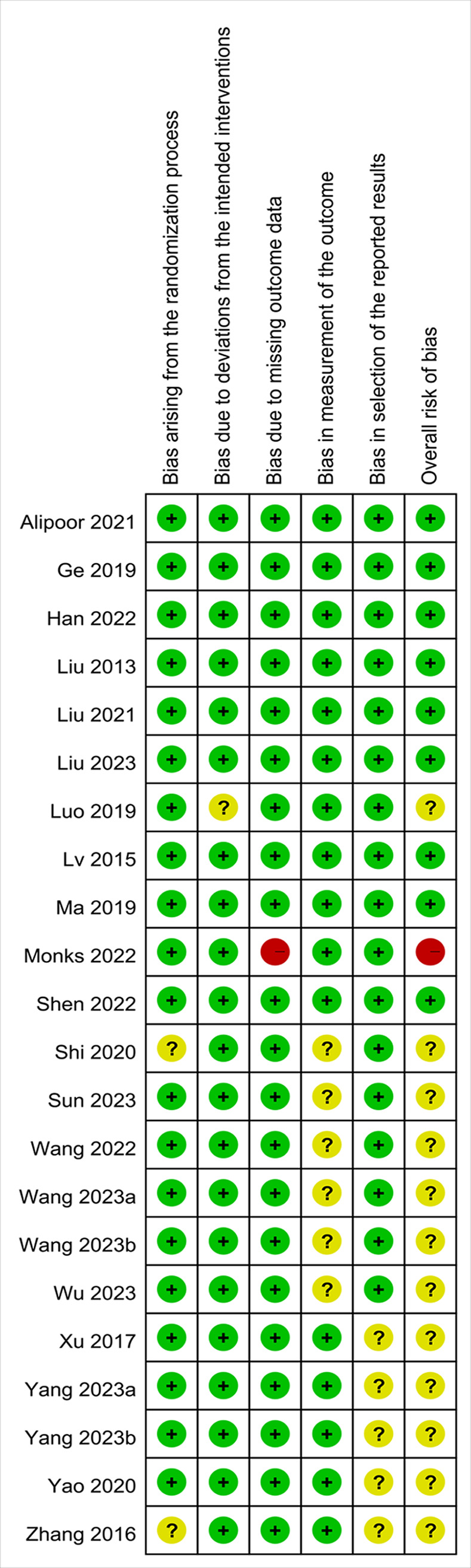
Risk of bias across 22 included studie.

For identification of PPDS, 19 studies used varying cutoff scores on the EPDS, including scores of 9 [17–19, 33, 34], 10 [35, 41, 44, 46, 50], 12 [40], and 13 [38, 39, 42, 43, 45, 48, 49]. However, two studies did not provide the details regarding the cutoff scores for identifying PPDS [36, 47]. One study used the Postpartum Depression Screening Scale with a cutoff of >75 for identifying PPDS [32]. The time points for PPDS assessment ranged from 1–180 days following CS. Regarding geographical locations, most studies were conducted in China (n = 20) [17–19, 32–36, 38, 39, 41–47, 49, 50], with the remaining studies from Iran (n = 1) [48] and the United States (n = 1) [40]. The risk of bias across studies is presented in Fig 2 and S4 Table. Regarding the overall risk of bias, nine [17, 18, 41, 43–48], twelve [19, 32–36, 38, 39, 42, 49, 50], and one [40] studies were deemed low, unclear, and high risk of bias, respectively.

### 3.3. Results of meta-analysis

#### 3.3.1. Primary outcome: PPDS incidence at 1- and 4–6-week follow-ups

**Fig 3:**
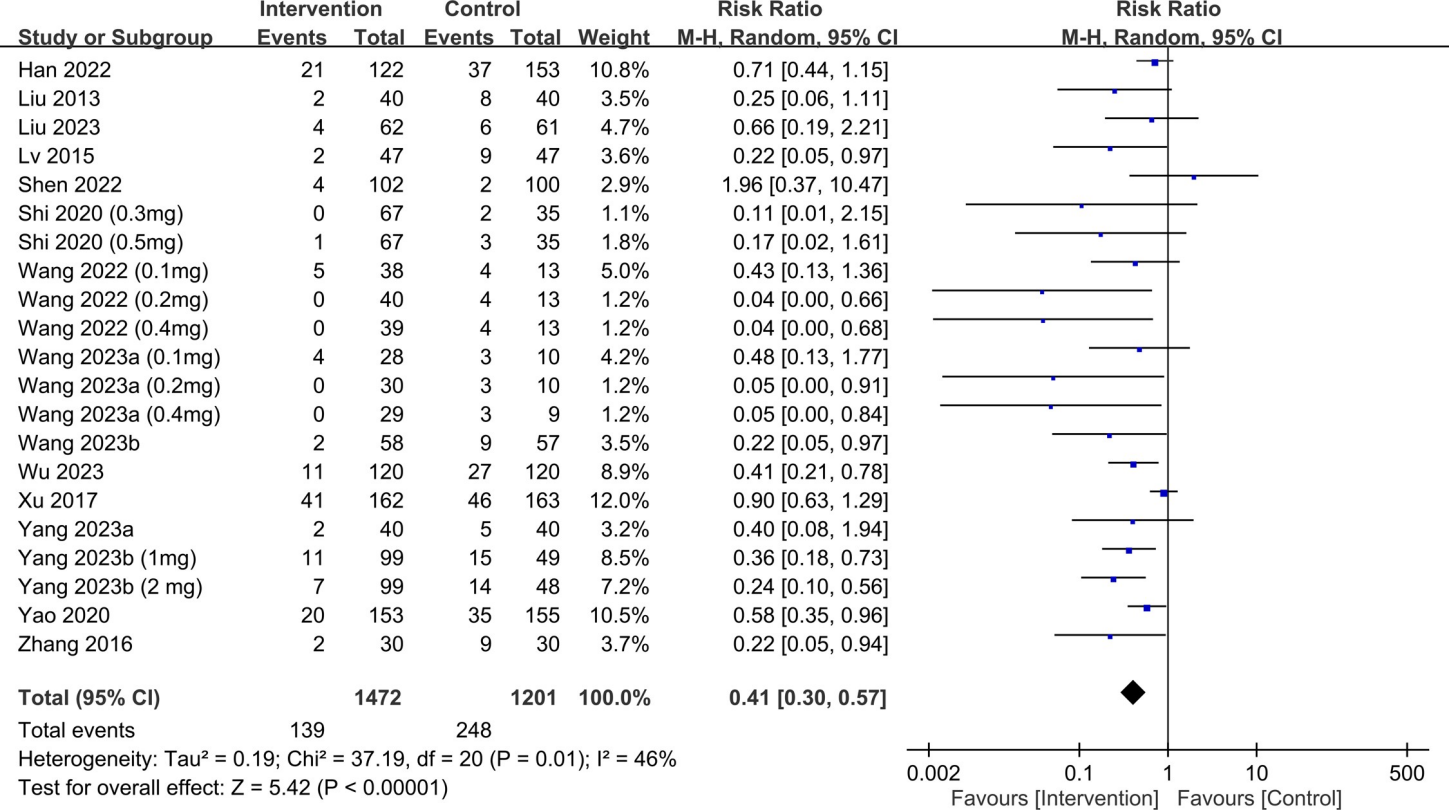
Forest plot showing the incidence of postpartum depression symptoms (PPDS) at 1 week. CI: confidence interval; M-H: Mantel–Haenszel.

**Fig 4:**
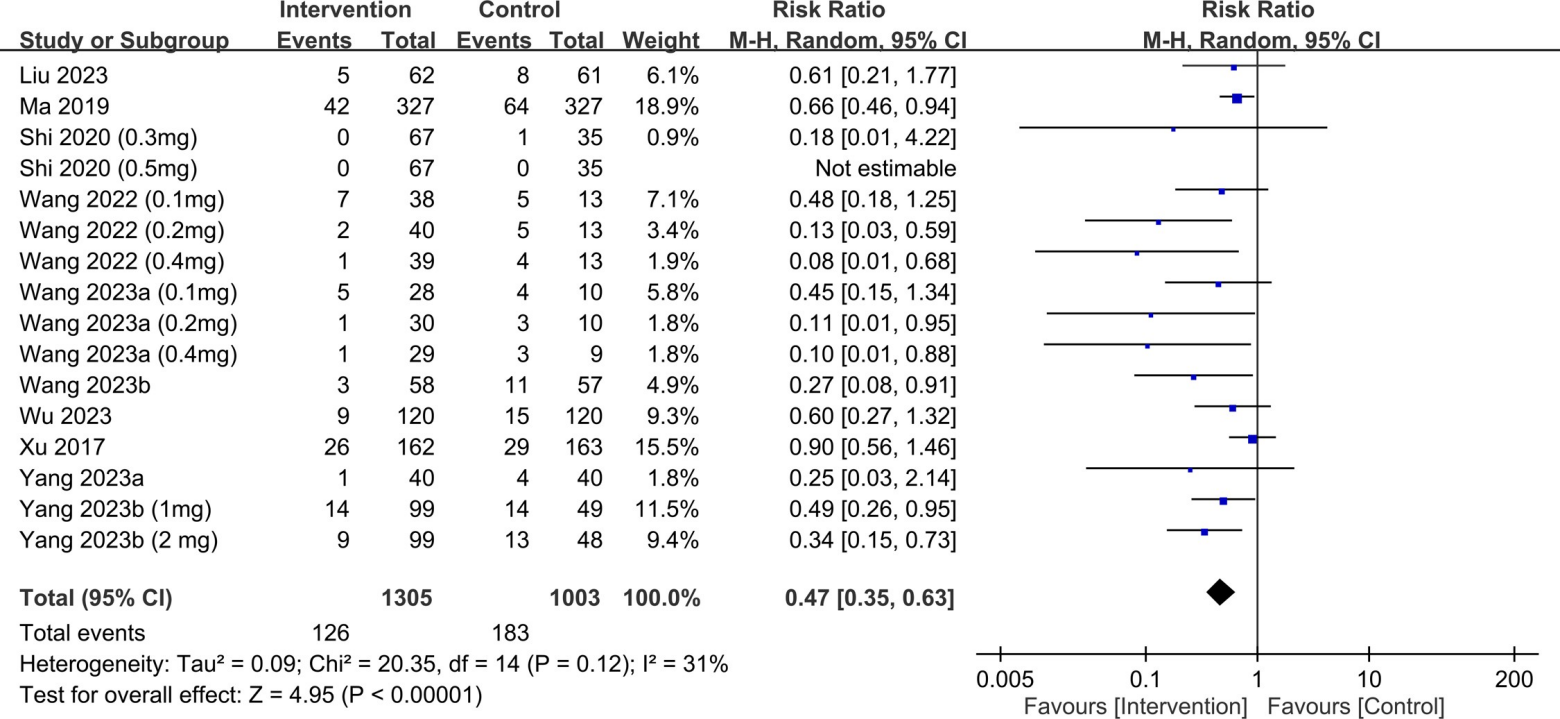
Forest plot showing the incidence of postpartum depression symptoms (PPDS) at 4−6 weeks. CI: confidence interval; M-H: Mantel–Haenszel.

**Fig 5:**
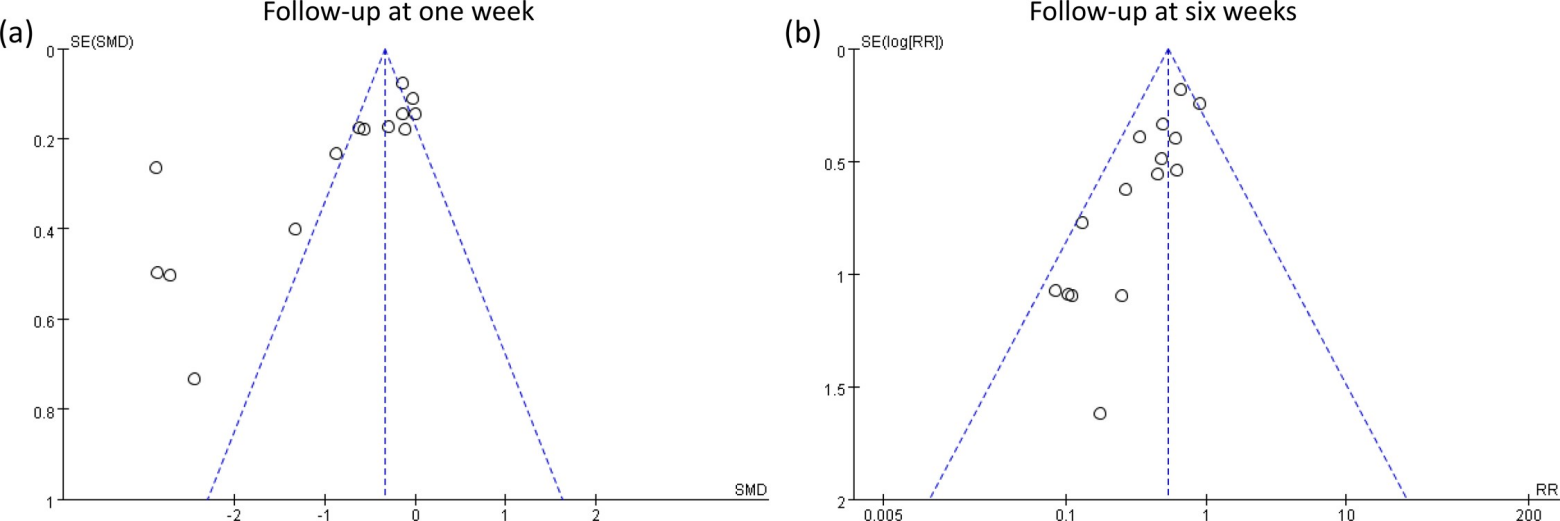
Funnel plot assessing publication bias in studies evaluating postpartum depression (PPD) risk. Each point represents an individual study, plotted based on the effect size (horizontal axis) versus the standard error (vertical axis). The funnel plot’s asymmetry, evident in the uneven distribution of studies on either side of the mean effect size (solid vertical line), suggests the potential for significant publication bias. (a): Risk of PPD at one-week follow-up; (b) Risk of PPD at 4-6-week follow-up.

**Fig 6:**
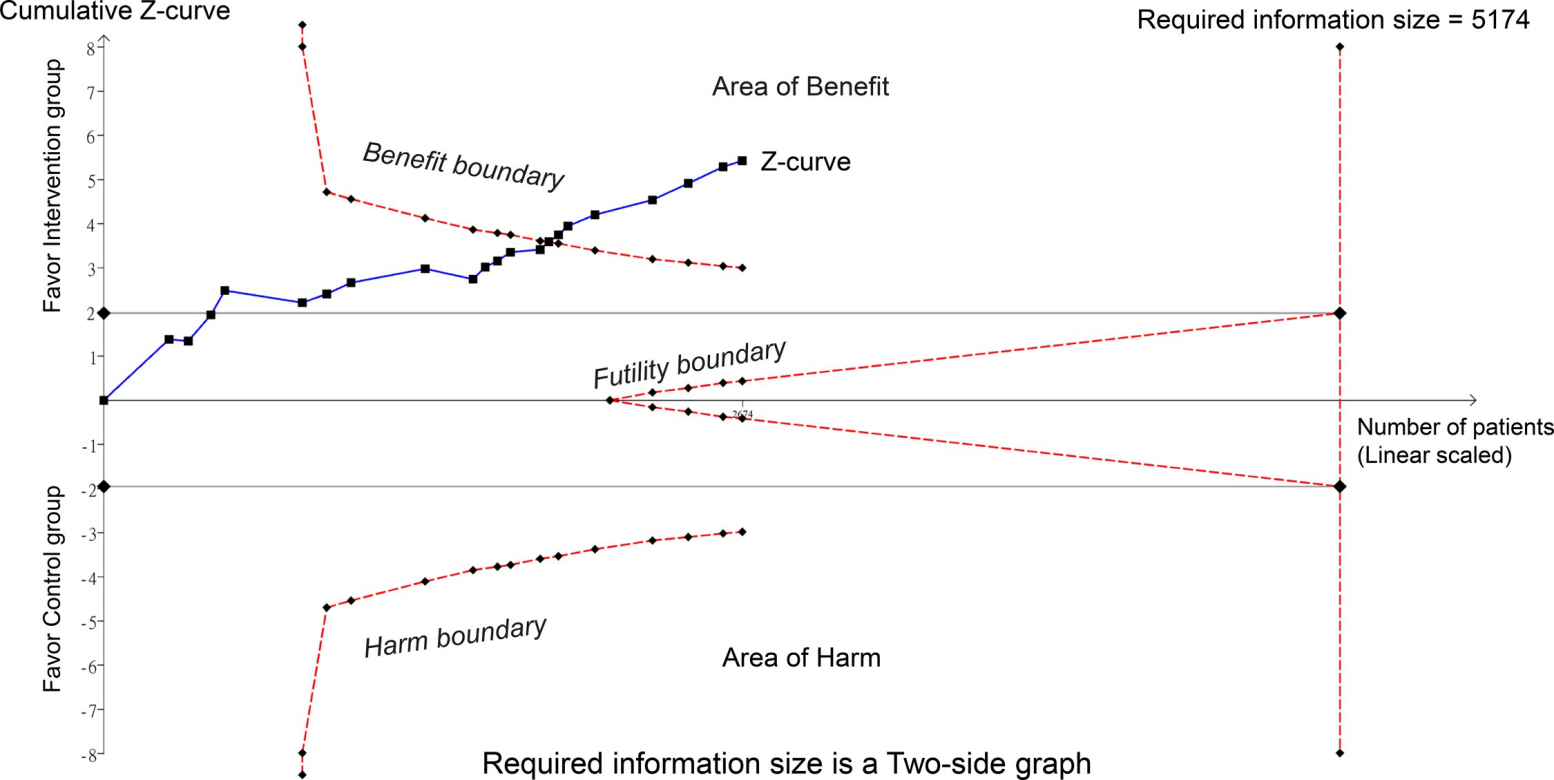
Trial sequential analysis (TSA) for postpartum depression (PPD) risk at 1-week follow-up. As illustrated, the z-curve crosses the TSA boundary, indicating that the accumulated evidence has achieved the requisite level of statistical significance and robustness, thereby affirming the reliability of the intervention observed. Additional trials are unlikely to alter this conclusion, underlining the sufficiency of the current evidence. The z-curve (blue line) represents the cumulative z-score as each trial is added sequentially. The TSA boundary (i.e., benefit boundary) signifies the adjusted threshold for statistical significance, taking into account repetitive testing on accumulating data.

**Fig 7:**
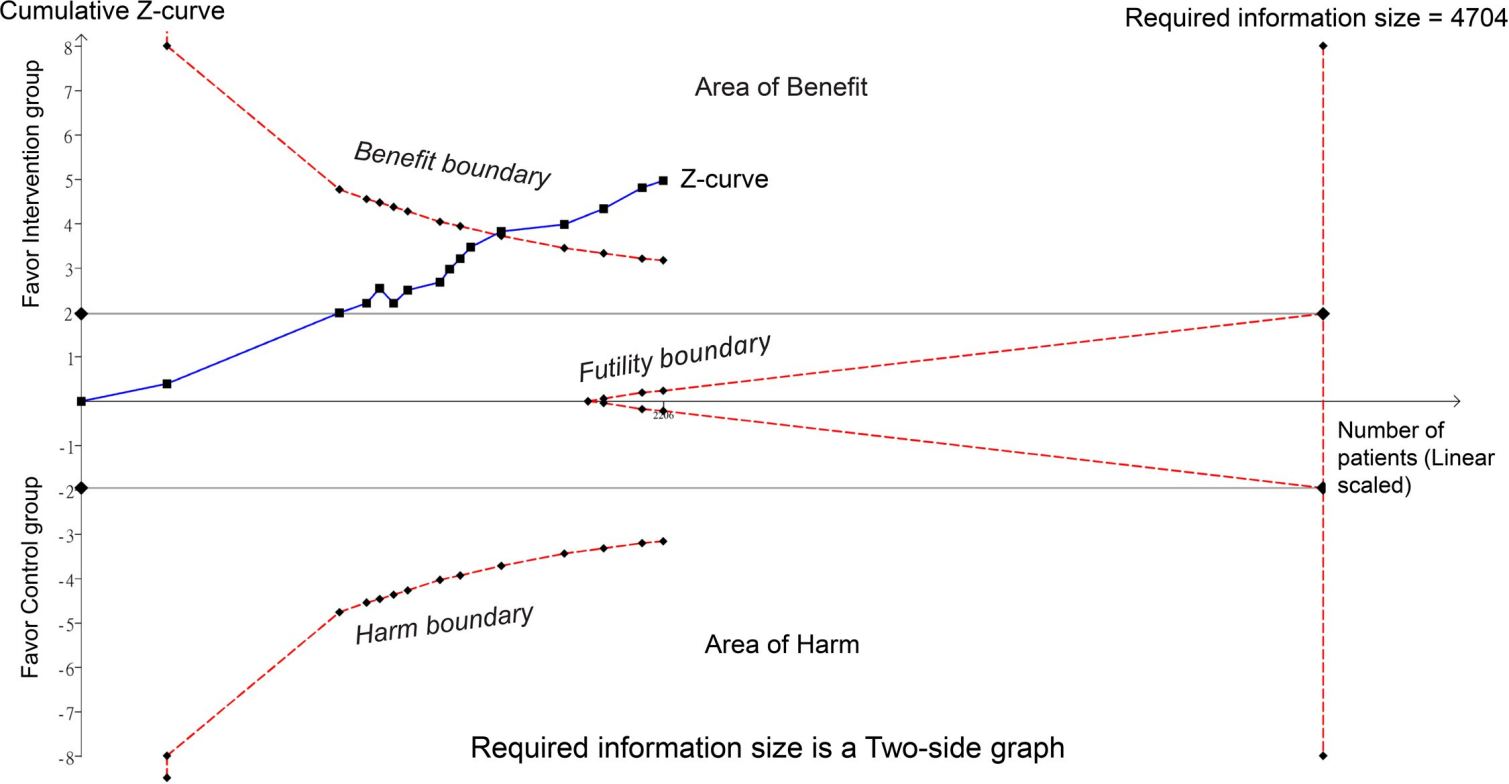
Trial sequential analysis (TSA) for postpartum depression (PPD) risk at 4–6-week follow-up. As illustrated, the z-curve crosses the TSA boundary, indicating that the accumulated evidence has achieved the requisite level of statistical significance and robustness, thereby affirming the reliability of the intervention observed. Additional trials are unlikely to alter this conclusion, underlining the sufficiency of the current evidence. The z-curve (blue line) represents the cumulative z-score as each trial is added sequentially. The TSA boundary (i.e., benefit boundary) signifies the adjusted threshold for statistical significance, taking into account repetitive testing on accumulating data.

raw data used for analysis is available in S5 Table. The meta-analysis of 15 trials with 21 pairwise comparisons (datasets) showed a significantly lower PPDS incidence with ketamine/esketamine use than that of a placebo at 1-week follow-up (RR, 0.41; 95% CI, 0.3–0.57; p < 0.00001; I^2^ = 46%; 2,673 participants) (Fig 3) [18, 19, 32–36, 38, 39, 41, 44–46, 50]. Consistently, patients receiving ketamine/esketamine were at a lower PPDS incidence at 4–6-week follow-up (RR, 0.47; 95% CI, 0.35–0.63; p < 0.00001; I^2^ = 31%; 2,308 participants) (Fig 4) [17, 19, 34–36, 38, 39, 45, 50]. Sensitivity analyses for the two outcomes were consistent, indicating the robustness of the findings. Visual inspection of the funnel plot showed asymmetry, suggesting a potential publication bias (Fig 5). The results from Egger’s test indicated a significant risk of publication bias for studies assessing PPDS at 1- (p = 0.00007) and 4–6-week follow-ups (p = 0.0001). To assess the robustness and reliability of the accumulated evidence, TSA was performed. For both PPDS incidence at 1- (Fig 6) and 4–6-week (Fig 7) follow-ups, the results showed that the Z-curve surpassed the TSA boundary, demonstrating that the evidence was not only statistically significant but also robust.

The findings of subgroup analyses are shown in Table 2. Ketamine/esketamine use was effective in preventing PPDS at 1-week postpartum, regardless of the time of administration, type of regimens (e.g., ketamine vs. esketamine), and total dosage (e.g., <0.5 vs. ≥0.5 mg/kg). Furthermore, no significant differences were noted between these subgroup analyses. However, regarding PPDS occurrence at 4–6-week postpartum, the postoperative use of ketamine/esketamine (RR: 0.33, 95% CI, 0.19–0.55) was more effective in preventing PPDS than its intraoperative use (RR: 0.67, 95% CI, 0.44–1.00) (Table 2). Additionally, although both ketamine and esketamine were effective in preventing PPDS occurrence at 4–6-week postpartum, esketamine (RR: 0.39, 95% CI, 0.29–0.53) use was more effective than that of ketamine (RR: 0.73, 95% CI, 0.55–0.97) (subgroup difference, p = 0.004) (Table 2). The ketamine/esketamine dosage did not impact the prophylactic efficacy against PPDS (subgroup difference, p = 0.07).

**Table 2:** Subgroup analysis on the incidence of postpartum depression symptoms (PPDS).

Outcomes or subgroups	*n* ^*†*^	Participants	Effect Estimate	I^2^ (%)	Subgroup difference, p-value
PPDS incidence at one week					
Time of administration					
Intraoperatively	9	1388	0.46 [0.27, 0.78]	50	0.49
Postoperatively	9	867	0.35 [0.20, 0.62]	44	
Type of ketamine					
Ketamine	7	1071	0.45 [0.26, 0.78]	51	0.67
Esketamine	14	1602	0.39 [0.26, 0.58]	37	
Total dosage⁋					
<0.5 mg	12	1404	0.40 [0.23, 0.70]	54	0.95
≥0.5 mg	9	1269	0.41 [0.29, 0.57]	16	
PPDS incidence at 4–6 weeks					
Time of administration					
Intraoperatively	5	1298	0.67 [0.44, 1.00]	30	0.03
Postoperatively	8	592	0.33 [0.19, 0.55]	16	
Type of ketamine					
Ketamine	4	1183	0.73 [0.55, 0.97]	0	0.004
Esketamine	12	1125	0.39 [0.29, 0.53]	0	
Total dosage⁋					
<0.5 mg	10	894	0.31 [0.17, 0.57]	49	0.07
≥0.5 mg	6	1414	0.57 [0.44, 0.75]	0	

†Datasets; PPDS: postpartum depression symptoms

⁋total dosage of ketamine/esketamine regardless of the time of administration

**Fig 8:**
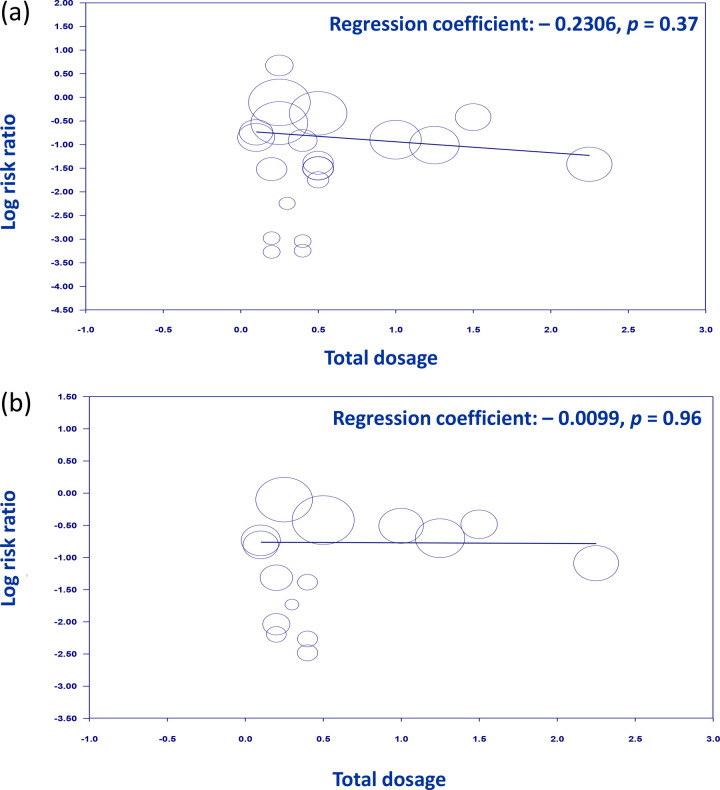
Meta-regression analysis of the influence of total dosage on postpartum depression (PPD) risk at (a) 1- and (b) 4–6-week follow-ups. The plot illustrates individual studies as points, positioned according to their respective total dosages (horizontal axis) and corresponding effect sizes measured as the risk of PPD (vertical axis). The sizes of the points represent the weight of each study in the meta-analysis. The solid line represents the regression line, indicating the relationship between total dosage and PPD risk.

To investigate the potential influence of total dosage on PPDS incidence at 1- and 4–6-week follow-ups, a meta-regression analysis was performed (Fig 8). The findings showed no significant association between the total dosage and the PPDS incidence at both 1- (regression coefficient, −0.2306; p = 0.37) (Fig 8A) and 4–6-week (regression coefficient, −0.0099; p = 0.96) (Fig 8B) follow-ups. This indicates that, within the studied dosage ranges, the dose administered does not affect the likelihood of developing PPDS during these particular postpartum intervals.

#### 3.3.2. Secondary outcomes: difference in depression-related scores and risk of other adverse events

**Fig 9:**
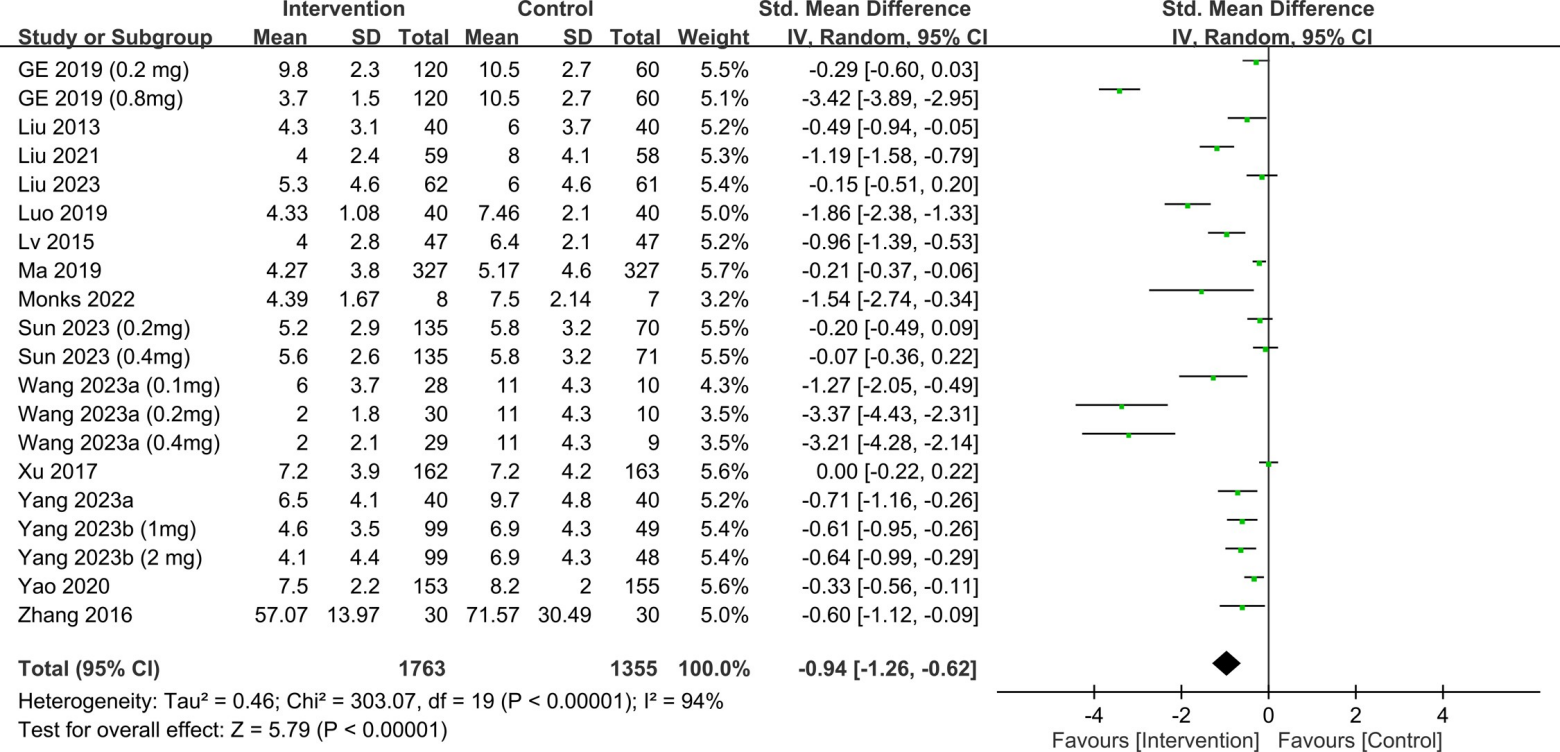
Forest plot showing differences in depression scores at 1-week follow-up. CI: confidence interval; IV: invariance; Std: standardized.

**Fig 10:**
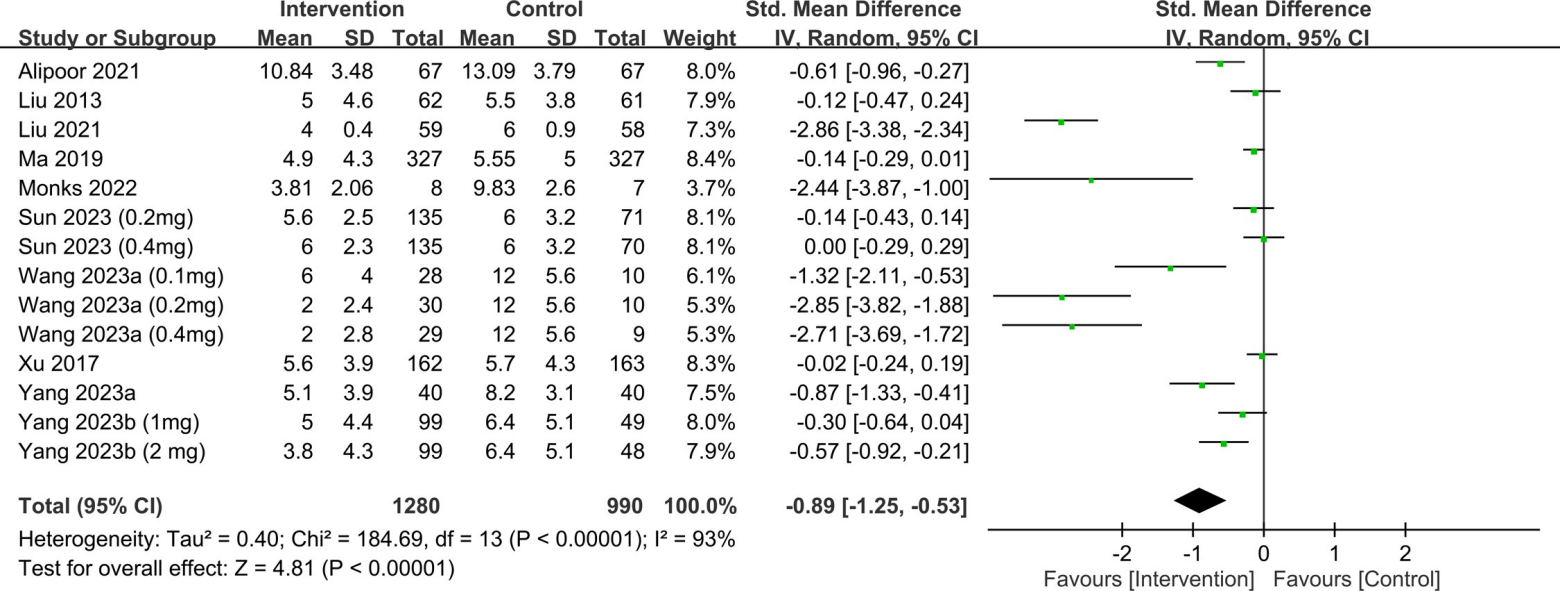
Forest plot showing differences in depression scores at 4–6-week follow-up. CI: confidence interval; IV: invariance; Std: standardized.

Participants receiving ketamine/esketamine had lower depression-related scores at 1- (SMD, −0.94; 95% CI, −1.26 to −0.62; p < 0.00001; I^2^ = 94%; 3,118 participants; sensitivity analysis: consistent) (Fig 9) [17, 19, 32–35, 38, 40–45, 47, 49] and 4–6-week (SMD, −0.89; 95% CI, −1.25 to −0.53; p < 0.00001; I^2^ = 93%; 2,270 participants; sensitivity analysis: consistent) (Fig 10) [17, 19, 34, 35, 38, 40, 43, 44, 48, 49] follow-ups. Visual inspection of the funnel plot showed asymmetry, suggesting a potential publication bias on outcomes at 1- (S1 Fig) and 4–6-week (S2 Fig) follow-ups.

Meta-analysis showed a significant association between ketamine/esketamine use and the risk of hallucinations (RR, 4.77; 95% CI, 1.39–16.44; p = 0.01; I^2^ = 0%; 2,140 participants; sensitivity analysis: consistent) (S3 Fig) and dizziness (RR, 1.36; 95% CI, 1.02–1.81; p = 0.04; I^2^ = 0%; 3,579 participants; sensitivity analysis: inconsistent) (S4 Fig). Nevertheless, no significant differences were observed in the risk of PONV (RR, 0.92; 95% CI, 0.64–1.32; p = 0.65; I^2^ = 48%; 3,966 participants; sensitivity analysis: consistent) (S5 Fig), drowsiness, pruritus, and headache (S6 Fig) between both groups. Subgroup analyses revealed that ketamine was linked to an increased likelihood of experiencing hallucinations and PONV compared to the control group (S3–S5 Figs). In contrast, esketamine did not demonstrate an elevated risk for these adverse events (S3–S5 Figs). Visual inspection of the funnel plot suggested a low risk of publication bias on outcomes including risks of PONV (S7 Fig) and dizziness (S8 Fig).

#### 3.3.3. Certainty of evidence

The evidence for depression risk at 1 week and 4–6 weeks, as well as depression-related scores at 1 week and 4–6 weeks, was rated as moderate certainty (Table 3). The reasons for downgrading included inconsistency and potential risk of publication bias. For dizziness, PONV, drowsiness, pruritus, and headache, the evidence was rated as moderate certainty, with imprecision being the reason for downgrading. Certainty was rated as high for hallucination outcomes. Overall, the certainty of the evidence ranged from moderate to high across the outcomes, indicating a reasonable level of confidence in the effect estimates (Table 3).

**Table 3:** Certainty of evidence based on the Grading of Recommendations Assessment, Development and Evaluation (GRADE) approach.

Outcomes	n†	Participants	Certainty assessment (Domains)					Effect size [95% CI]	Certainty
			A	B	C	D	E		
Depression risk 1 week	21	2673	NS	NS	NS	NS	S	RR 0.41 [0.30, 0.57]	⨁⨁⨁◯ Moderate
Depression risk 4–6 weeks	16	2308	NS	NS	NS	NS	S	RR 0.47 [0.35, 0.63]	⨁⨁⨁◯ Moderate
Depression-related scores 1 week	20	3118	NS	S	NS	NS	S	SMD -0.94 [-1.26, -0.62]	⨁⨁⨁◯ Moderate^†^
Depression-related scores 4–6 weeks	14	2270	NS	S	NS	NS	S	SMD -0.89 [-1.25, -0.53]	⨁⨁⨁◯ Moderate^†^
Dizziness	18	3579	NS	NS	NS	S	NS	RR 1.36 [1.02, 1.81]	⨁⨁⨁◯ Moderate
Hallucination	8	2140	NS	NS	NS	NS	-	RR 4.77 [1.39, 16.44]	⨁⨁⨁⨁ High
PONV	25	3966	NS	NS	NS	S	NS	RR 0.92 [0.64, 1.32]	⨁⨁⨁◯ Moderate
Drowsiness	4	730	NS	NS	NS	S	-	RR 1.49 [0.80, 2.78]	⨁⨁⨁◯ Moderate
Pruritus	6	364	NS	NS	NS	S	-	RR 0.66 [0.21, 2.12]	⨁⨁⨁◯ Moderate
Headache	4	1025	NS	NS	NS	S	-	RR 1.39 [0.54, 3.53]	⨁⨁⨁◯ Moderate

A: risk of bias; B: Inconsistency; C: Indirectness; D: Imprecision; E: publication bias; NS: not serious; S: serious; RR: Risk Ratio; SMD: Standardized Mean Difference; CI: Confidence Interval; PONV: Postoperative Nausea and Vomiting; †number of studies or datasets. †Certainty of evidence was upgraded due to large effect size

## 4. Discussion

### 4.1. Summary of findings

Based on our analysis of 22 RCTs, ketamine/esketamine administration significantly reduced PPDS incidence compared with a placebo during 1- and 4–6-week follow-ups; however, it was also correlated with an increased incidence of hallucinations and dizziness. Consistently, participants administered with ketamine/esketamine reported lower depression-related scores at 1 and 4–6-week assessments. Despite an asymmetrical funnel plot suggesting possible publication bias, the robustness of the presented evidence is reinforced by the TSA findings. Subgroup analyses showed that the preventive effect of ketamine/esketamine on PPDS within the first postpartum week was consistent, irrespective of administration timing, ketamine type, and total dosage (e.g., <0.5 vs. ≥0.5 mg/kg). In contrast, for the 4–6-week postpartum period, ketamine/esketamine proved more effective in preventing PPDS when administered postoperatively rather than intraoperatively. Furthermore, although ketamine and esketamine were successful in reducing PPDS occurrences at the 4–6-week follow-up, esketamine demonstrated superior efficacy. The ketamine/esketamine dosage was not a significant factor in its prophylactic effectiveness against PPDS (subgroup difference, p = 0.07).

### 4.2. Ketamine/esketamine use and PPDS incidence

Our findings are partially consistent with the results of Li et al. [20], which also showed a significantly reduced PPDS incidence at 1 week following perioperative ketamine administration. In contrast to the study by Li et al. [20], which did not assess PPDS incidence at 4–6 weeks between the ketamine and control groups, our findings suggest that ketamine/esketamine retains its preventive effect against PPDS at the 4–6-week follow-up. Additionally, the benefits of ketamine/esketamine in reducing PPDS occurrence were corroborated by the TSA findings, an aspect not investigated in previous meta-analyses [20]. This finding is significant as a previous study has indicated that proactive prevention before PPDS onset significantly reduces depressive symptoms in women predisposed to PPD development [52]. For individuals at a high risk of PPD including those with previous history of depression [53], such prevention strategy may be considered. We identified potential risks of publication bias on our primary outcomes. Considering that the majority of these studies were conducted in Asian countries, coupled with the publication bias concern, more studies are needed to ascertain the efficacy of ketamine/esketamine in preventing PPDS in clinical settings.

### 4.3. Subgroup analysis

The interesting findings were that although timing, type of ketamine, and dosage did not impact 1-week PPDS incidence, administration time and type of ketamine did affect PPDS incidence at 4–6 weeks. Postoperative administration appeared more effective than intraoperative usage in reducing 4–6-week PPDS incidence. Although the exact reason for this finding remains elusive, it could be that postoperative application ensures prolonged exposure to ketamine/esketamine during a period susceptible to PPDS onset. Additionally, although both drugs were successful, esketamine (RR: 0.39) was found to be more effective than ketamine (RR: 0.73) in preventing 4 to 6-week PPDS (subgroup difference, p = 0.004) (Table 2). Esketamine’s greater potency, potentially due to enhanced AMPA receptor activation [16, 54], may offer benefits in PPDS prevention. The overall ketamine/esketamine dosage did not significantly impact the PPDS preventive effect at either follow-up, implying that the minimum effective dose may be achieved even at lower dosages. This has useful clinical implications in optimizing ketamine/esketamine dosage to balance efficacy and side effect risks. Further studies can help refine administration protocols for optimal longer-term PPDS prophylaxis.

### 4.4. EPDS: Characteristics and limitations

The EPDS, the predominant screening tool for PPD, comprises a 10-item self-report questionnaire, prompting women to reflect on their feelings over the past week [55, 56]. Questions are scored from 0–3, with an overall score range of 0–30, and it typically requires approximately 5 min to complete. Scores of 12/13 and 9/10 indicate “probable” and “possible” depression, respectively [57]. In our current meta-analysis, we prioritized prevention of PPDS as the primary outcome over differences in EPDS scores. This decision was based on the understanding that although depression severity generally corresponds to the total score beyond a certain cutoff, the cumulative EPDS score may not necessarily align with depression severity when the score remains below this threshold (e.g., a cutoff of 12). Significant heterogeneity was observed in the depression-related scores, indicating that these outcomes might be inappropriate for evaluating the effectiveness of ketamine/esketamine.

### 4.5. Side effects

Ketamine is known to induce dissociative psychedelic effects by acting as an NMDA receptor antagonist [58]. Our meta-analysis showed that ketamine/esketamine use was associated with a significantly higher risk of experiencing hallucinations and dizziness than that of placebo controls. The estimated four-fold increased risk of hallucinations is consistent with other studies, indicating that perceptual disturbances are common side effects of these agents [34, 59]. The temporary psychotomimetic effects may be distressing and negatively impact patient experience. Nevertheless, strategies that help mitigate these side effects are available, including careful patient selection, setting, dose adjustment, and providing education and reassurance. Considering the transient nature of these adverse effects, the risks may be tolerable for several patients to gain the antidepressant benefits, especially under medical supervision.

### 4.6. Novelty and strengths of the current study

A recent meta-analysis by Li et al.[60] similarly explored the effectiveness of ketamine and esketamine in preventing PPDS. Despite some overlap in subject matter, our study offers significant novel contributions. First, our meta-analysis included a larger dataset (22 studies involving 3,463 participants) than the meta-analysis by Li et al. [60] (14 studies involving 2,916 participants), enhancing the robustness of our findings. Second, our exclusive focus on RCTs, as opposed to the recent meta-analysis [60] that also included a retrospective study (i.e., 13 RCTs and one retrospective study), further strengthens evidence quality. Methodologically, we employed TSA, a technique that was not used in the study by Li et al. [60], to validate the reliability and conclusiveness of our primary outcomes. Moreover, we assessed the risk of bias using the updated RoB 2.0, providing a more nuanced evaluation than the Cochrane risk of bias tool used by Li et al. [60]. These methodological enhancements ensured that our study adds new insights and offers a more comprehensive and reliable analysis.

### 4.7. Limitation

Although this meta-analysis provides compelling evidence supporting ketamine/esketamine use for PPDS prevention, some limitations should be acknowledged. First, a major limitation was the lack of long-term follow-up beyond 6-week postpartum across the included studies. As such, whether the preventive effect is maintained over several months remains uncertain. Second, most trials were conducted in Asian countries, particularly in China. To support the generalizability of the findings to Caucasian and other populations, further studies in more ethnically diverse cohorts are required. Third, although subgroup analyses were performed, meta-regressions assessing contributors such as comorbidities, breastfeeding status, and social support were not performed owing to limitations in the reported data. Confounder effects of these variables on PPDS incidence cannot be excluded. Fourth, the included studies utilized varying cut-off scores on depression screening tools, primarily the EPDS, to identify patients with PPDS. Heterogeneity in the cut-off scores could impact the reliability of the pooled risk estimates. Finally, publication bias remains a concern, as suggested by the funnel plots’ asymmetry. However, the use of TSA helped confirm that the meta-analysis results were robust despite this bias. Overall, although the current meta-analysis makes a significant contribution, future studies should address these limitations through longer follow-up, more diverse populations, controlling confounders, and testing reproducibility.

### 4.8. Conclusion

Our results indicate that the perioperative use of ketamine/esketamine reduces PPDS occurrence at 1 and 4–6 weeks after surgery. Although the risk of transient hallucinations and dizziness increased with the use of ketamine/esketamine, no major adverse events were reported. As most of the studies were conducted in Asian countries and considering concerns on publication bias, more extensive studies across diverse populations to further validate our findings are urgently needed.

## Supporting information

S1 Fig Funnel plot showing depression-related scores at 1-week follow-up. (PDF)

S2 Fig Funnel plot showing depression-related scores at 4–6-week follow-up. (PDF)

S3 Fig Forest plot showing risk of hallucination. (PDF)

S4 Fig Forest plot showing risk of dizziness. (PDF)

S5 Fig Forest plot showing risk of postoperative nausea and vomiting. (PDF)

S6 Fig Forest plot showing risk of other adverse events. (PDF)

S7 Fig Funnel plot showing risk of postoperative nausea and vomiting. (PDF)

S8 Fig Funnel plot showing risk of dizziness. (PDF)

S1 Table PRISMA 2020 checklist. (DOCX)

S2 Table Search strategies for MEDLINE. (DOCX)

S3 Table Studies included and excluded. (DOCX)

S4 Table Risk of bias for each study. (DOCX)

S5 Table Raw data used in current meta-analysis. (DOCX)
