# Supplementary figures and images for: Perioperative administration of sub-anesthetic ketamine/esketamine for preventing postpartum depression symptoms: A trial sequential meta-analysis
Source: PLoS One. 2024 Nov 18;19(11):e0310751. doi: 10.1371/journal.pone.0310751 (PMC11573214; doi:10.1371/journal.pone.0310751)

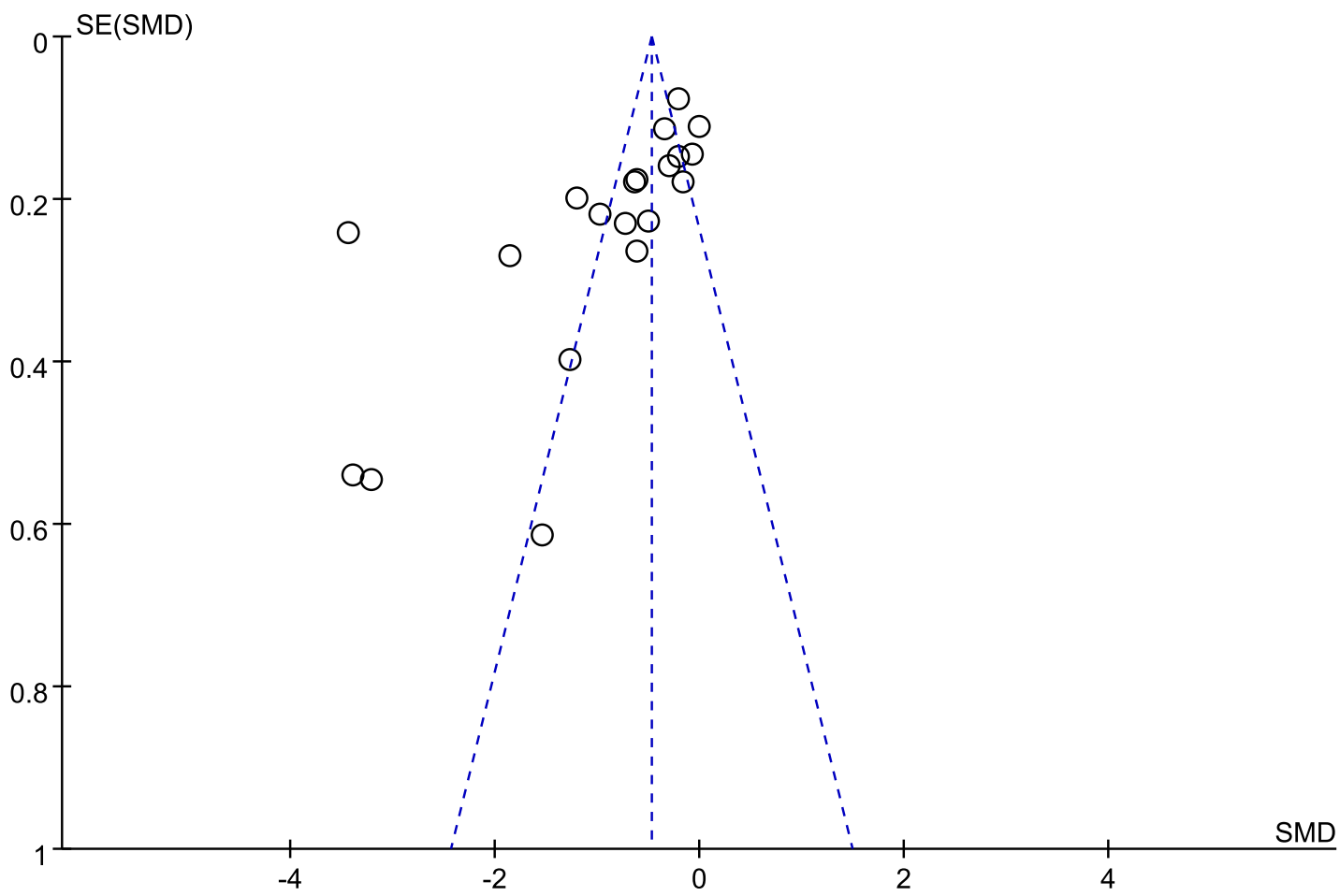

Supplement: S1 Fig — (PDF) [file pone.0310751.s001.pdf]

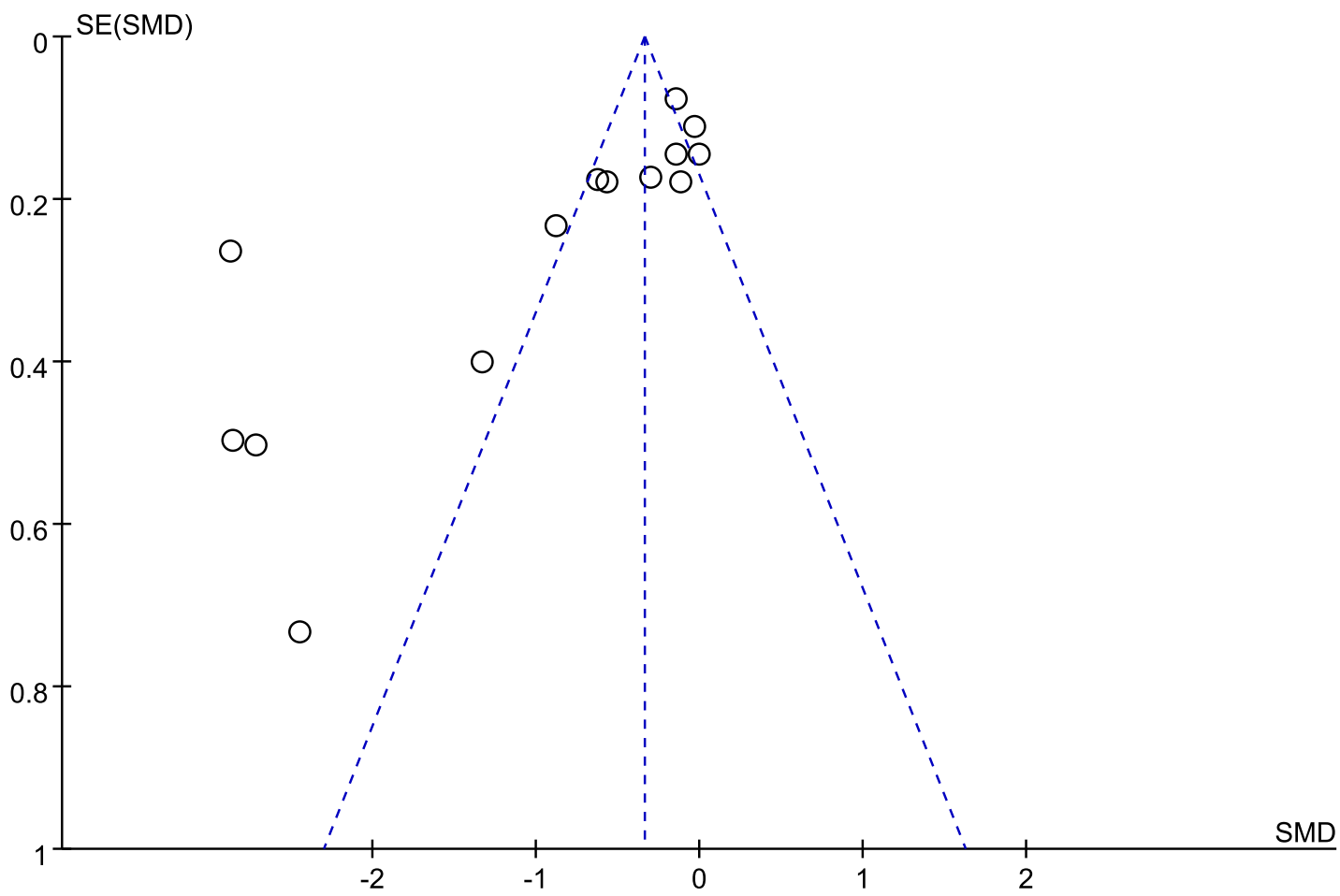

Supplement: S2 Fig — (PDF) [file pone.0310751.s002.pdf]

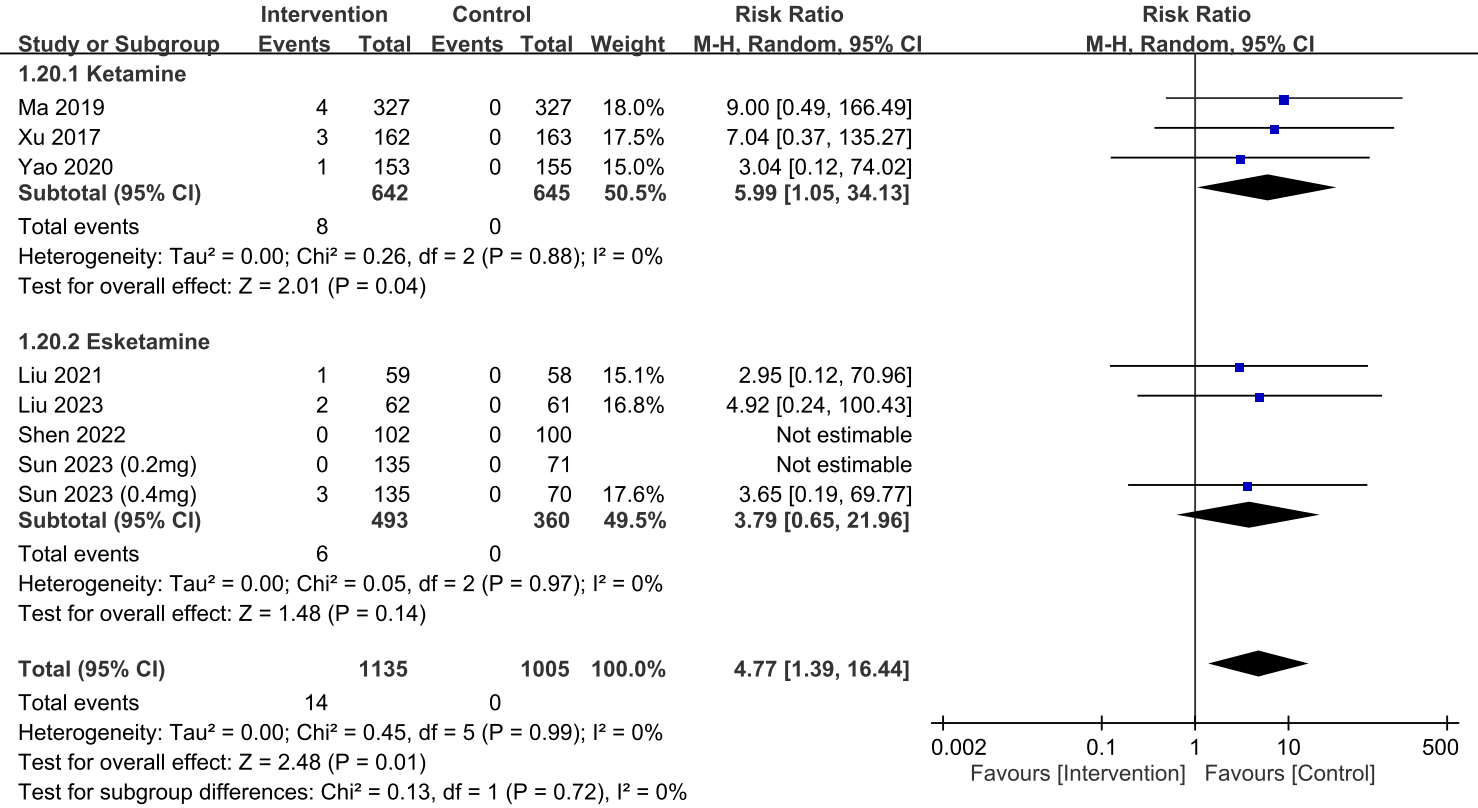

Supplement: S3 Fig — (PDF) [file pone.0310751.s003.pdf]

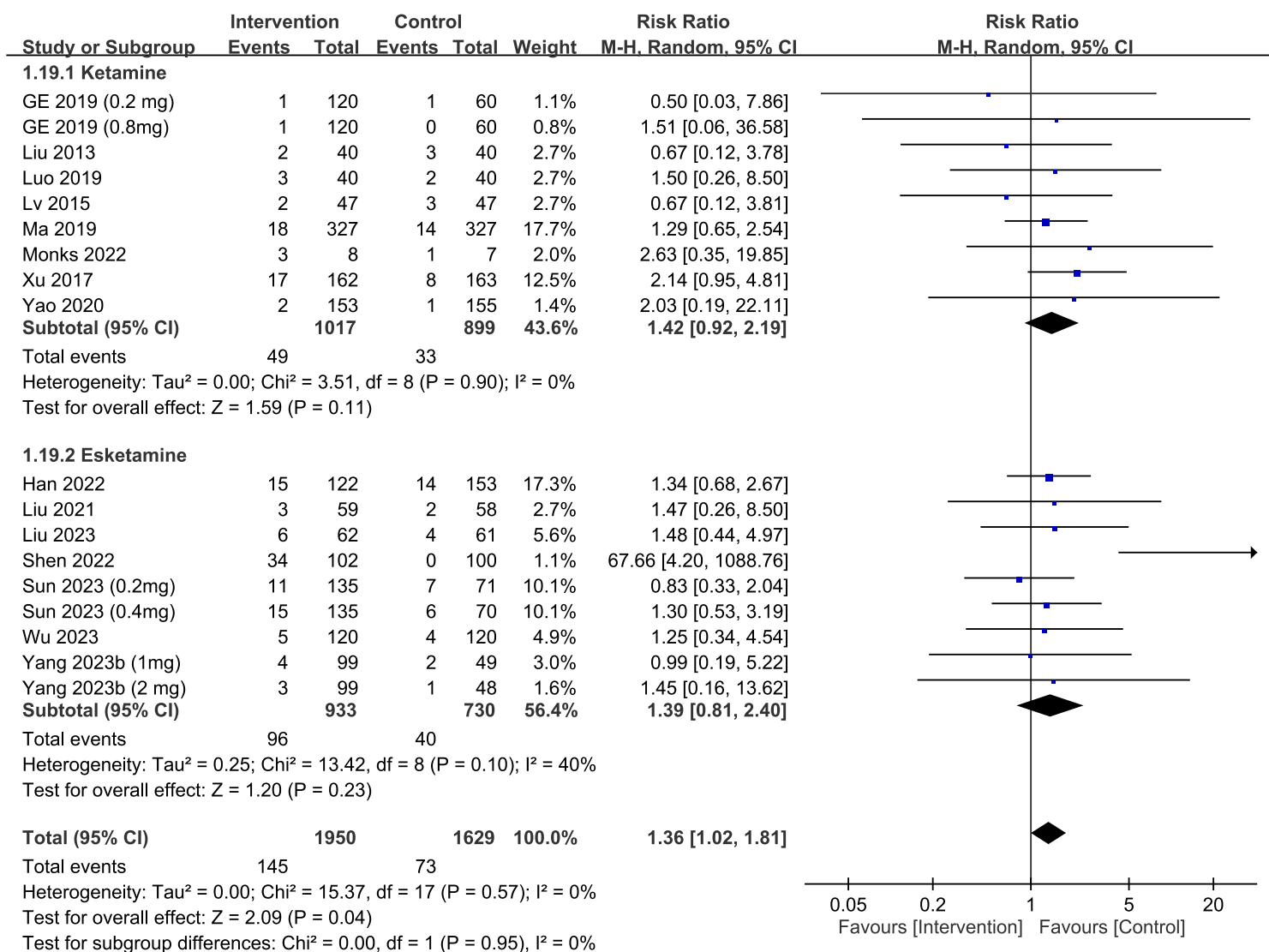

Supplement: S4 Fig — (PDF) [file pone.0310751.s004.pdf]

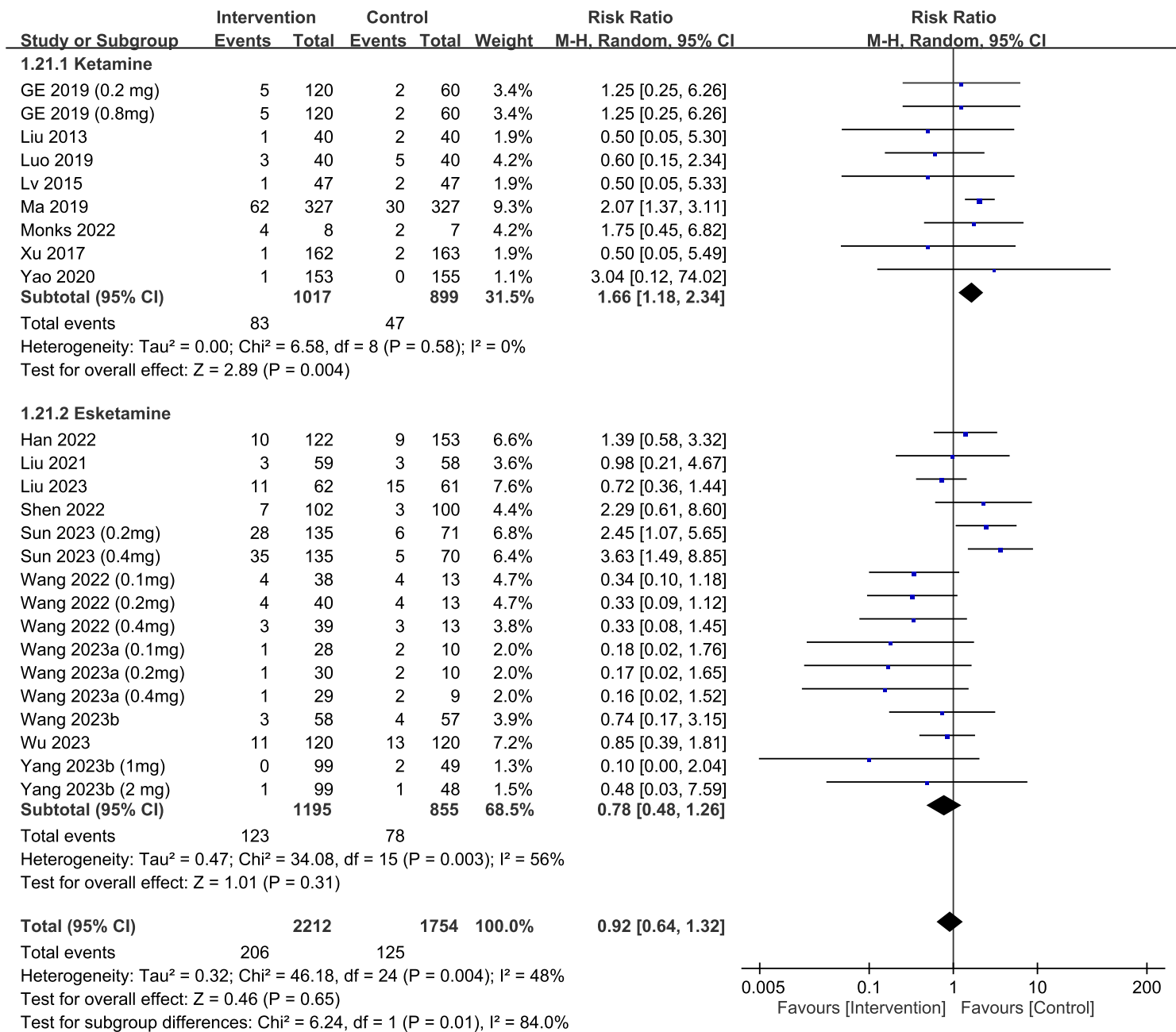

Supplement: S5 Fig — (PDF) [file pone.0310751.s005.pdf]

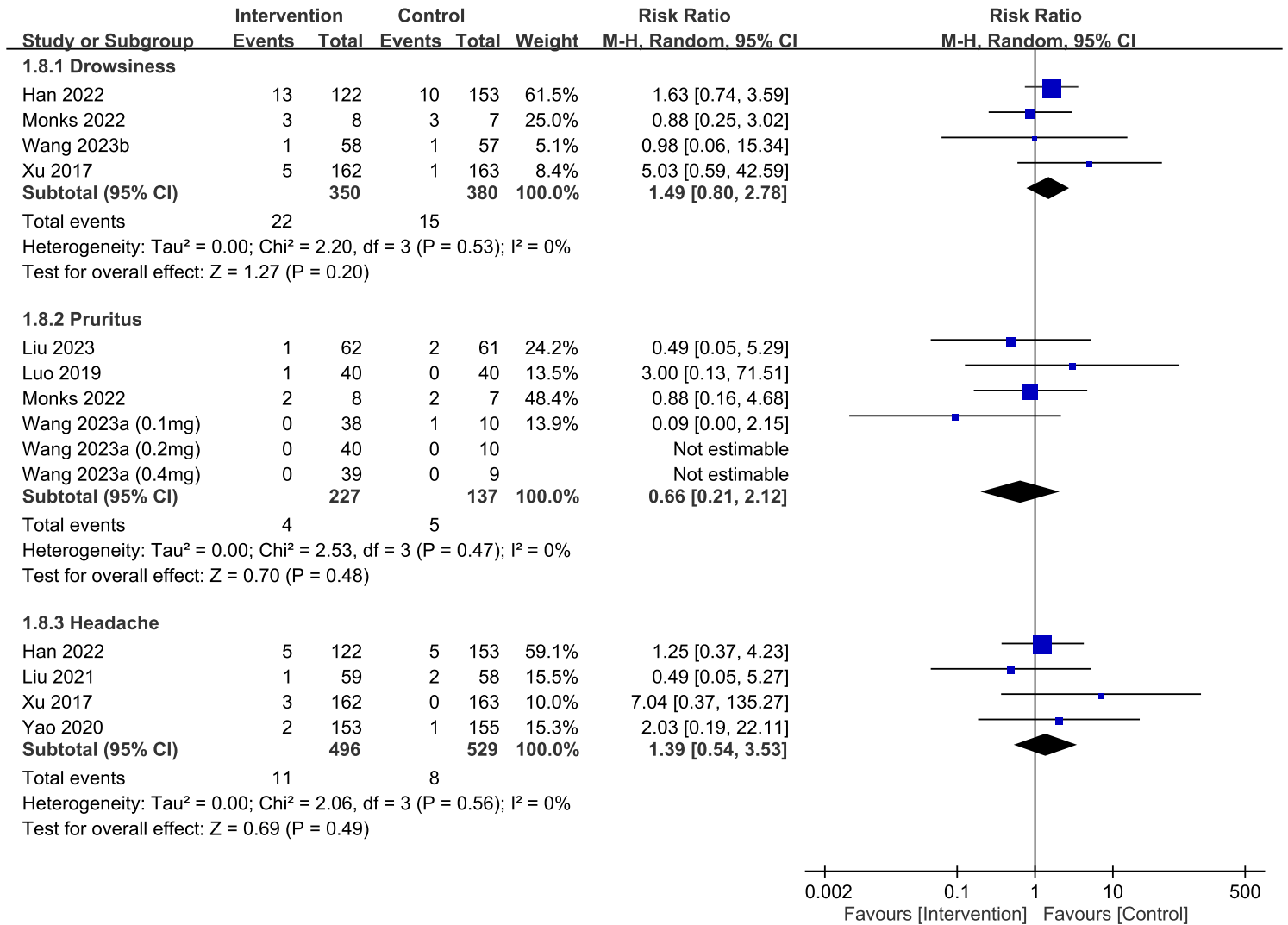

Supplement: S6 Fig — (PDF) [file pone.0310751.s006.pdf]

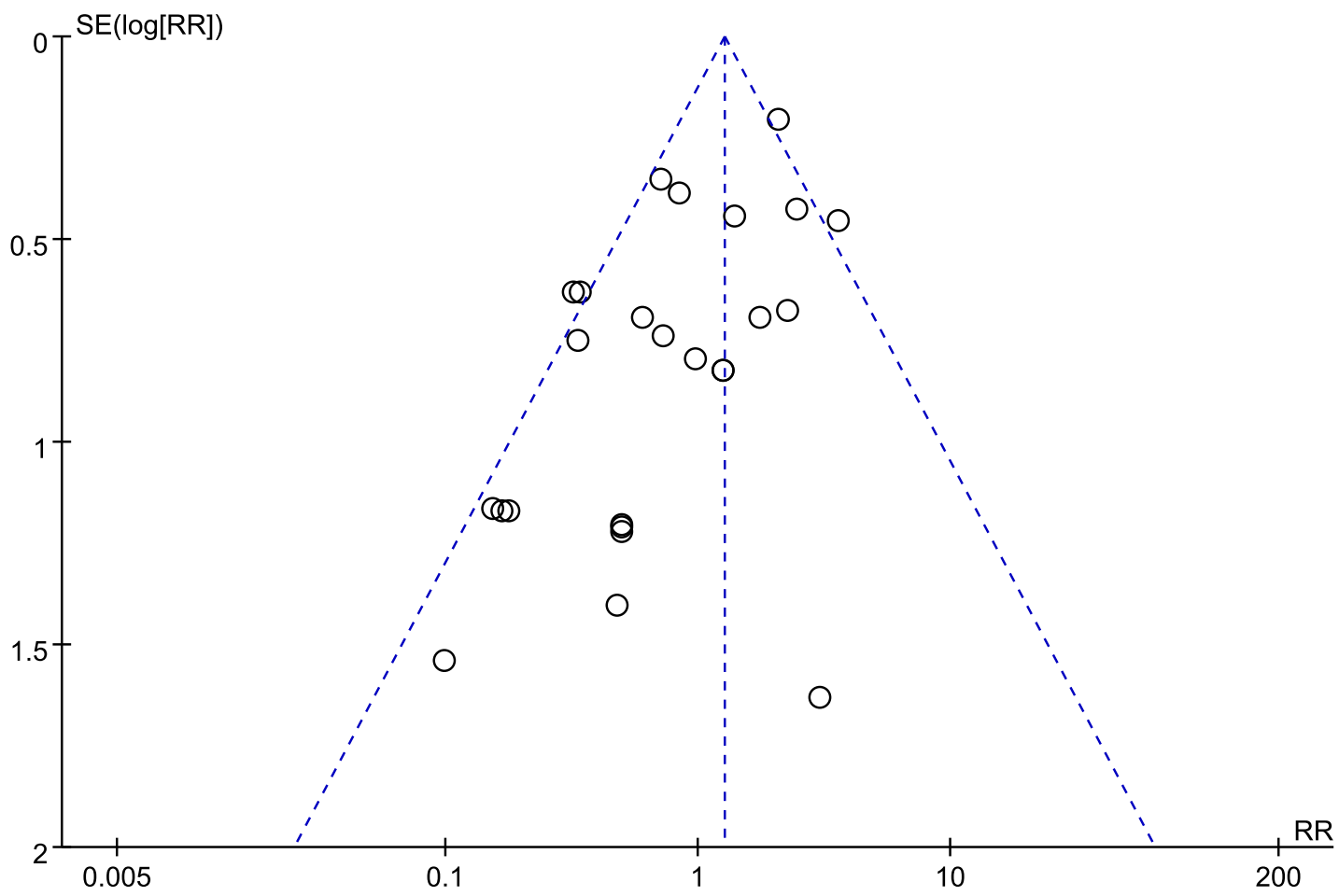

Supplement: S7 Fig — (PDF) [file pone.0310751.s007.pdf]

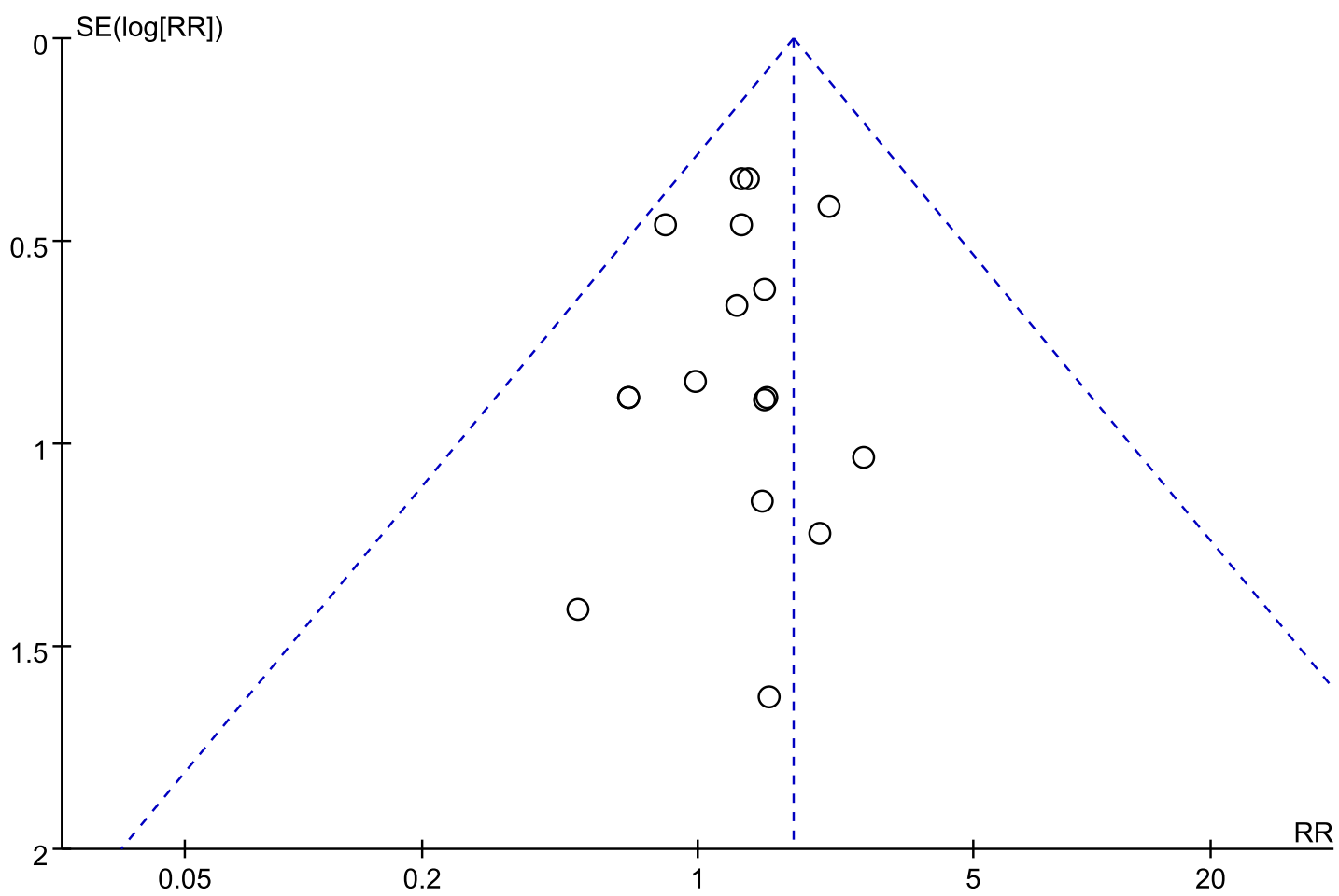

Supplement: S8 Fig — (PDF) [file pone.0310751.s008.pdf]
