# Supplementary material for: Perioperative administration of sub-anesthetic ketamine/esketamine for preventing postpartum depression symptoms: A trial sequential meta-analysis
Source: PLoS One. 2024 Nov 18;19(11):e0310751. doi: 10.1371/journal.pone.0310751 (PMC11573214; doi:10.1371/journal.pone.0310751)
Supplement: S2 Table — (DOCX) [file pone.0310751.s010.docx]

**Supplemental Table 2.** Search strategies for MEDLINE

| 1 | ("cesarean section*" or "C-Sections" or "Postcesarean Section").mp. |
| --- | --- |
| 2 | exp "Cesarean Section"/ |
| 3 | ("Ketamine" or "Esketamine" or "Sketamine" or "Spravato" or "Kataved" or "L-Ketamine" or "Ketalar" or "Ketaset" or "Ketanest" or "Calipsol" or "Kalipsol" or "Calypsol").mp. |
| 4 | exp "Ketamine"/ |
| 5 | ("postpartum depression" or "Postnatal Depression" or "Post-Partum Depression" or "Post-Partum Dysphoria" or "Depression" or "Depressive syndrome").mp. |
| 6 | exp "Depression, Postpartum"/ |
| 7 | (1 or 2) and (3 or 4) and (5 or 6) |
| 8 | 7 and (((randomized controlled trial or controlled clinical trial).pt. or randomi*ed.ab. or placebo.ab. or drug therapy.fs. or randomly.ab. or trial.ab. or groups.ab.) not (exp animals/ not humans.sh.)) |
