# Supplementary material for: Perioperative administration of sub-anesthetic ketamine/esketamine for preventing postpartum depression symptoms: A trial sequential meta-analysis
Source: PLoS One. 2024 Nov 18;19(11):e0310751. doi: 10.1371/journal.pone.0310751 (PMC11573214; doi:10.1371/journal.pone.0310751)
Supplement: S3 Table — (DOCX) [file pone.0310751.s011.docx]

**Supplemental Table 3.** Studies included and excluded

| **Included studies** | |
| --- | --- |
|  | - Alipoor, M., Loripoor, M., Kazemi, M., Farahbakhsh, F., & Sarkoohi, A. (2021). The effect of ketamine on preventing postpartum depression. J Med Life, 14(1), 87-92. doi:10.25122/jml-2020-0116 - Ge, J., Sun, X., Jiang, X., & Song, J. (2019). Effect of ketamine on analgesia and postpartum depression after cesarean section. J Xuzhou Med Univ, 39(11), 810-814. - Han, Y., Li, P., Miao, M., Tao, Y., Kang, X., & Zhang, J. (2022). S-ketamine as an adjuvant in patient-controlled intravenous analgesia for preventing postpartum depression: a randomized controlled trial. BMC Anesthesiol, 22(1), 49. doi:10.1186/s12871-022-01588-7 - Liu, Q. R., Zong, Q. K., Ding, L. L., Dai, H. Y., Sun, Y., Dong, Y. Y., . . . Yang, J. J. (2023). Effects of perioperative use of esketamine on postpartum depression risk in patients undergoing cesarean section: A randomized controlled trial. J Affect Disord, 339, 815-822. doi:10.1016/j.jad.2023.07.103 - Liu, X., Hu, X., Zhang, W., Ling, C., Lin, J., & Du, S. (2013). Effect of pre-administration of low-dose ketamine on Edinburgh Postnatal Depression Scale scores in women after cesarean section. Guangdong Medical Journal, 34(12), 1917-1919. - Liu, Y., & Li, X. (2021). Effects of esketamine combined with hydromorphone on analgesia and postpartum depression after cesarean section. Shandong Medical Journal, 61(19), 84-87. - Luo, Z. (2019). Effect of sufentanil combined with low-dose ketamine applied to analgesia after cesarean section. Guangxi Medical Journal, 41(14), 1774-1777. - Lv, L. (2015). Effect of maternal pre-injection of low dose ketamine on postpartum depression score in cesarean section. Medical Recapitulate, 21(24), 4570-4571. - Ma, J. H., Wang, S. Y., Yu, H. Y., Li, D. Y., Luo, S. C., Zheng, S. S., . . . Duan, K. M. (2019). Prophylactic use of ketamine reduces postpartum depression in Chinese women undergoing cesarean section(✰). Psychiatry Res, 279, 252-258. doi:10.1016/j.psychres.2019.03.026 - Monks, D. T., Palanisamy, A., Jaffer, D., Singh, P. M., Carter, E., & Lenze, S. (2022). A randomized feasibility pilot-study of intravenous and subcutaneous administration of ketamine to prevent postpartum depression after planned cesarean delivery under neuraxial anesthesia. BMC Pregnancy Childbirth, 22(1), 786. doi:10.1186/s12884-022-05118-8 - Shen, J., Song, C., Lu, X., Wen, Y., Song, S., Yu, J., & Sun, J. (2022). The effect of low-dose esketamine on pain and post-partum depression after cesarean section: A prospective, randomized, double-blind clinical trial. Front Psychiatry, 13, 1038379. doi:10.3389/fpsyt.2022.1038379 - Shi, J., Yang, Y. J., Tian, Y. M., & Wang, Z. Y. (2020). Study on the prophylactic effect of low-dose ketamine on postpartum depression after cesarean section. Journal of Modern Medicine & Health, 36(18), 2956-2958. - Sun, S., Xu, Z., & Sun, H. (2023). Effects of intravenous infusion of esketamine on analgesia and postpartum antidepressant after cesarean section. Journal of Hainan Medical University, 29(18), 1395-1400. - Wang, W., Xu, H., Chen, Q., Ling, B., Lyu, J., & Yu, W. (2023). Effects of different doses of esketamine on analgesia and postpartum depression after cesarean section. Clinical Anesthesiology, 39(5), 501-505. - Wang, W., Ling, B., Chen, Q., Xu, H., Lv, J., & Yu, W. (2023). Effect of pre-administration of esketamine intraoperatively on postpartum depression after cesarean section: A randomized, double-blinded controlled trial. Medicine (Baltimore), 102(9), e33086. doi:10.1097/md.0000000000033086 - Wang, W., Xu, H., Ling, B., Chen, Q., Lv, J., & Yu, W. (2022). Effects of esketamine on analgesia and postpartum depression after cesarean section: A randomized, double-blinded controlled trial. Medicine (Baltimore), 101(47), e32010. doi:10.1097/md.0000000000032010 - Wu, W., & Wang, L. (2023). Effects of esketamine combined with nalbuphine for patient-controlled intravenous analgesia on their analgesia, recovery quality, and postpartum depression after cesarean section. Chinese Journal of Family Planning, 31(8), 1812-1817. - Xu, Y., Li, Y., Huang, X., Chen, D., She, B., & Ma, D. (2017). Single bolus low-dose of ketamine does not prevent postpartum depression: a randomized, double-blind, placebo-controlled, prospective clinical trial. Arch Gynecol Obstet, 295(5), 1167-1174. doi:10.1007/s00404-017-4334-8 - Yang, F., Wei, L., Wu, Y., Shen, M., Zan, L., & Su, H. (2023). Effects of intervention with esketamine after cesarean section on postpartum depression, Treg cell percentage, and IL-10 expression. Journal of Nantong University (Medical Sciences), 43(3), 253-256. - Yang, S. Q., Zhou, Y. Y., Yang, S. T., Mao, X. Y., Chen, L., Bai, Z. H., . . . et al. (2023). Effects of different doses of esketamine intervention on postpartum depressive symptoms in cesarean section women: a randomized, double-blind, controlled clinical study. J Affect Disord, 339, 333‐341. doi:10.1016/j.jad.2023.07.007 - Yao, J., Song, T., Zhang, Y., Guo, N., & Zhao, P. (2020). Intraoperative ketamine for reduction in postpartum depressive symptoms after cesarean delivery: A double-blind, randomized clinical trial. Brain and Behavior, 10(9), e01715. doi:https://dx.doi.org/10.1002/brb3.1715 - Zhang, Q., Liu, Z., & Wang, X. (2016). Effect of low-dose ketamine on prevention of depression after cesarean section. Journal of North Sichuan Medical College, 31(4), 502-505. |
| **Reasons for exclusion** | **Studies excluded** |
| No data for analysis | Sheng, F., Chen, L., Du, M., & Fang, R. (2022). Effect of Esketamine on Postoperative Analgesia and Postpartum Depression after Cesarean Section. Pharmacy Today, 32(2), 150-152. |
| Review article | Pang, L., Cui, M., Dai, W., Kong, J., Chen, H., & Wu, S. (2020). Can Intraoperative Low-Dose R,S-Ketamine Prevent Depressive Symptoms After Surgery? The First Meta-Analysis of Clinical Trials. Frontiers in Pharmacology, 11, 586104. doi:https://dx.doi.org/10.3389/fphar.2020.586104 |
| Review article | Liang, S., Du, Y., Jia, D., Tu, F., & Wang, J. (2022). Application of esketamine for postoperative analgesia in cesarean section: a meta-analysis. [Chinese]. *Chinese Journal of Evidence-Based Medicine, 22(6)*, 667-675. doi:https://dx.doi.org/10.7507/1672-2531.202202005 |
| Retrospective study | Lou, F., Wang, C., Dong, X., Jin, L., Chen, H., Lu, Y., & Yang, Z. (2023). Analysis of the Analgesic Effect, Emotion, and Safety of Esketamine in Cesarean Section Analgesia for Puerperae. *Alternative Therapies in Health & Medicine, 29*(7), 424-428. |
| Retrospective study | Wang, Y., Zhang, Q., Dai, X., Xiao, G., & Luo, H. (2022). Effect of low-dose esketamine on pain control and postpartum depression after cesarean section: a retrospective cohort study. *Ann Palliat Med, 11*(1), 45-57. doi:10.21037/apm-21-3343 |
| Conference abstract | Houthoff Khemlani, K., Jokinen, J., Kranke, P., & Schreiber, J. U. (2014). Hypnotic agents for induction of general anaesthesia in caesarean section patients: Systemic review and metaanalysis of randomized trials. *International Journal of Obstetric Anesthesia, 1)*, S15. doi:https://dx.doi.org/10.1016/j.ijoa.2014.03.011 |
| Conference abstract | Loripoor, M., Kazemi, M., & Alipoor, M. (2018). The effect of ketamine for general anesthesia in caesarean section on postpartum depression. *International Journal of Gynecology and Obstetrics, 143(Supplement 3)*, 289. doi:https://dx.doi.org/10.1002/ijgo.12582 |
| Conference abstract | Schyns-Van Den Berg, A. (2017). Post-cesarean section analgesia. *Regional Anesthesia and Pain Medicine, 42(5 Supplement 1)*, e47-e48. doi:https://dx.doi.org/10.1097/AAP.0000000000000656 |
| Conference abstract | Sng, B. L. (2014). Opiate-free post-cesarean section analgesia: Can it be achieved? Regional Anesthesia and Pain Medicine, 1), e87-e88. doi:https://dx.doi.org/10.1097/AAP.0000000000000142 |
| Review article | Chen-Li, D., Lui, L. M. W., Rosenblat, J. D., Lipsitz, O., Teopiz, K. M., Ho, R., . . . McIntyre, R. S. (2022). Ketamine as potential treatment for postpartum depression: A narrative review. *Annals of Clinical Psychiatry, 34(4)*, 264-274. doi:https://dx.doi.org/10.12788/acp.0082 |
| Review article | Jolly, A. S., Jain, P., & Sood, J. (2007). Ketamine-current uses and future perspectives. *Journal of Anaesthesiology Clinical Pharmacology, 23(2)*, 169-181. |
| Inclusion criteria not met | Adhikari, P., Subedi, A., Sah, B. P., & Pokharel, K. (2021). Analgesic effects of intravenous ketamine after spinal anaesthesia for non-elective caesarean delivery: a randomised controlled trial. BMJ open, 11(6), e044168. doi:10.1136/bmjopen-2020-044168 |
| Inclusion criteria not met | Alipoor, M., Loripoor, M., Kazemi, M., Farahbakhsh, F., & Sarkoohi, A. (2021). The effect of ketamine on preventing postpartum depression. Journal of medicine and life, 14(1), 87‐92. doi:10.25122/jml-2020-0116 |
| Inclusion criteria not met | Han, Y., Li, P., Miao, M., Tao, Y., Kang, X., & Zhang, J. (2022). S-ketamine as an adjuvant in patient-controlled intravenous analgesia for preventing postpartum depression: a randomized controlled trial. BMC anesthesiology, 22(1), 49. doi:10.1186/s12871-022-01588-7 |
| Conference abstract | Leykin, Y., Pellis, T., & Zannier, G. (2006). Thiopental--ketamine association and low dose priming with rocuronium for rapid sequence in duction of anaesthesia for elective cesareum section. Minerva Anestesiologica, 72(7-8), 683-688. |
