# Supplementary material for: Perioperative administration of sub-anesthetic ketamine/esketamine for preventing postpartum depression symptoms: A trial sequential meta-analysis
Source: PLoS One. 2024 Nov 18;19(11):e0310751. doi: 10.1371/journal.pone.0310751 (PMC11573214; doi:10.1371/journal.pone.0310751)
Supplement: S4 Table — (DOCX) [file pone.0310751.s012.docx]

**Supplemental table 4.** Risk of bias for each study

| **Study ID** | **D1** | **D2** | **D3** | **D4** | **D5** | **Overall risk of bias** |
| --- | --- | --- | --- | --- | --- | --- |
| Alipoor 2021 | Low | Low | Low | Low | Low | Low |
| Ge 2019 | Low | Low | Low | Low | Low | Low |
| Han 2022 | Low | Low | Low | Low | Low | Low |
| Liu 2013 | Low | Low | Low | Low | Low | Low |
| Liu 2021 | Low | Low | Low | Low | Low | Low |
| Liu 2023 | Low | Low | Low | Low | Low | Low |
| Luo 2019 | Low | Some concern | Low | Low | Low | Unclear |
| Lv 2015 | Low | Low | Low | Low | Low | Low |
| Ma 2019 | Low | Low | Low | Low | Low | Low |
| Monks 2022 | Low | Low | High | Low | Low | High |
| Shen 2022 | Low | Low | Low | Low | Low | Low |
| Shi 2020 | Some concern | Low | Low | Unclear | Low | Some concern |
| Sun 2023 | Low | Low | Low | Unclear | Low | Some concern |
| Wang 2022 | Low | Low | Low | Unclear | Low | Some concern |
| Wang 2023a | Low | Low | Low | Unclear | Low | Some concern |
| Wang 2023b | Low | Low | Low | Unclear | Low | Some concern |
| Wu 2023 | Low | Low | Low | Unclear | Low | Some concern |
| Xu 2017 | Low | Low | Low | Low | Some concern | Some concern |
| Yang 2023a | Low | Low | Low | Low | Some concern | Some concern |
| Yang 2023b | Low | Low | Low | Low | Some concern | Some concern |
| Yao 2020 | Low | Low | Low | Low | Some concern | Some concern |
| Zhang 2016 | Some concern | Low | Low | Low | Some concern | Some concern |

D1:Bias arising from the randomization process

D2: Bias due to deviations from the intended interventions

D3: Bias due to missing outcome data

D4: Bias in measurement of the outcome

D5: Bias in selection of the reported results
