# Supplementary material for: Perioperative administration of sub-anesthetic ketamine/esketamine for preventing postpartum depression symptoms: A trial sequential meta-analysis
Source: PLoS One. 2024 Nov 18;19(11):e0310751. doi: 10.1371/journal.pone.0310751 (PMC11573214; doi:10.1371/journal.pone.0310751)
Supplement: S5 Table — (DOCX) [file pone.0310751.s013.docx]

**Supplemental table 5.** Raw data from the studies that was eligible to be included in the review **(**Name of data extractors: Kuo-Chuan Hung and Chia-Li Kao; date of data extraction: November 23, 2023**).**

| Study | outcomes | events | Total | events | total |  |  |
| --- | --- | --- | --- | --- | --- | --- | --- |
| Liu 2023 | risk 6w | 5 | 62 | 8 | 61 |  |  |
| Ma 2019 | risk 6w | 42 | 327 | 64 | 327 |  |  |
| Shi 2020 (0.3mg) | risk 6w | 0 | 67 | 1 | 35 |  |  |
| Shi 2020 (0.5mg) | risk 6w | 0 | 67 | 0 | 35 |  |  |
| Wang 2022 (0.1mg) | risk 6w | 7 | 38 | 5 | 13 |  |  |
| Wang 2022 (0.2mg) | risk 6w | 2 | 40 | 5 | 13 |  |  |
| Wang 2022 (0.4mg) | risk 6w | 1 | 39 | 4 | 13 |  |  |
| Wang 2023a (0.1mg) | risk 6w | 5 | 28 | 4 | 10 |  |  |
| Wang 2023a (0.2mg) | risk 6w | 1 | 30 | 3 | 10 |  |  |
| Wang 2023a (0.4mg) | risk 6w | 1 | 29 | 3 | 9 |  |  |
| Wang 2023b | risk 6w | 3 | 58 | 11 | 57 |  |  |
| Wu 2023 | risk 6w | 9 | 120 | 15 | 120 |  |  |
| Xu 2017 | risk 6w | 26 | 162 | 29 | 163 |  |  |
| Yang 2023a | risk 6w | 1 | 40 | 4 | 40 |  |  |
| Yang 2023b (1mg) | risk 6w | 14 | 99 | 14 | 49 |  |  |
| Yang 2023b (2 mg) | risk 6w | 9 | 99 | 13 | 48 |  |  |
|  |  |  |  |  |  |  |  |
|  |  |  |  |  |  |  |  |
|  |  |  |  |  |  |  |  |
| Study | outcomes | events | Total | events | total |  |  |
| Han 2022 | risk 1w | 21 | 122 | 37 | 153 |  |  |
| Liu 2013 | risk 1w | 2 | 40 | 8 | 40 |  |  |
| Liu 2023 | risk 1w | 4 | 62 | 6 | 61 |  |  |
| Lv 2015 | risk 1w | 2 | 47 | 9 | 47 |  |  |
| Shen 2022 | risk 1w | 4 | 102 | 2 | 100 |  |  |
| Shi 2020 (0.3mg) | risk 1w | 0 | 67 | 2 | 35 |  |  |
| Shi 2020 (0.5mg) | risk 1w | 1 | 67 | 3 | 35 |  |  |
| Wang 2022 (0.1mg) | risk 1w | 5 | 38 | 4 | 13 |  |  |
| Wang 2022 (0.2mg) | risk 1w | 0 | 40 | 4 | 13 |  |  |
| Wang 2022 (0.4mg) | risk 1w | 0 | 39 | 4 | 13 |  |  |
| Wang 2023a (0.1mg) | risk 1w | 4 | 28 | 3 | 10 |  |  |
| Wang 2023a (0.2mg) | risk 1w | 0 | 30 | 3 | 10 |  |  |
| Wang 2023a (0.4mg) | risk 1w | 0 | 29 | 3 | 9 |  |  |
| Wang 2023b | risk 1w | 2 | 58 | 9 | 57 |  |  |
| Wu 2023 | risk 1w | 11 | 120 | 27 | 120 |  |  |
| Xu 2017 | risk 1w | 41 | 162 | 46 | 163 |  |  |
| Yang 2023a | risk 1w | 2 | 40 | 5 | 40 |  |  |
| Yang 2023b (1mg) | risk 1w | 11 | 99 | 15 | 49 |  |  |
| Yang 2023b (2 mg) | risk 1w | 7 | 99 | 14 | 48 |  |  |
| Yao 2020 | risk 1w | 20 | 153 | 35 | 155 |  |  |
| Zhang 2016 | risk 1w | 2 | 30 | 9 | 30 |  |  |
|  |  |  |  |  |  |  |  |
|  |  |  |  |  |  |  |  |
|  |  |  |  |  |  |  |  |
|  |  |  |  |  |  |  |  |
| Study | outcomes | mean | sd | total | mean | sd | total |
| GE Jianlin 2019 (0.2 mg) | mean 1w | 9.8 | 2.3 | 120 | 10.5 | 2.7 | 60 |
| GE Jianlin 2019 (0.8mg) | mean 1w | 3.7 | 1.5 | 120 | 10.5 | 2.7 | 60 |
| Liu 2013 | mean 1w | 4.3 | 3.1 | 40 | 6 | 3.7 | 40 |
| Liu 2021 | mean 1w | 4. 9 | 2.4 | 59 | 8. 8 | 4.1 | 58 |
| Liu 2023 | mean 1w | 5.3 | 4.6 | 62 | 6.0 | 4.6 | 61 |
| Luo 2019 | mean 1w | 4.33 | 1.08 | 40 | 7.46 | 2.1 | 40 |
| Lv 2015 | mean 1w | 4 | 2.8 | 47 | 6.4 | 2.1 | 47 |
| Ma 2019 | mean 1w | 4.27 | 3.8 | 327 | 5.17 | 4.6 | 327 |
| Monks 2022 | mean 1w | 4.39 | 1.67 | 8 | 7.50 | 2.14 | 7 |
| Sun 2023 (0.2mg) | mean 1w | 5.2 | 2.9 | 135 | 5.8 | 3.2 | 70 |
| Sun 2023 (0.4mg) | mean 1w | 5.6 | 2.6 | 135 | 5.8 | 3.2 | 71 |
| Wang 2023a (0.1mg) | mean 1w | 6. 4 | 3.7 | 28 | 11. 7 | 4.3 | 10 |
| Wang 2023a (0.2mg) | mean 1w | 2. 1 | 1.8 | 30 | 11. 7 | 4.3 | 10 |
| Wang 2023a (0.4mg) | mean 1w | 2. 5 | 2.1 | 29 | 11. 7 | 4.3 | 9 |
| Xu 2017 | mean 1w | 7.2 | 3.9 | 162 | 7.2 | 4.2 | 163 |
| Yang 2023a | mean 1w | 6.5 | 4.1 | 40 | 9.7 | 4.8 | 40 |
| Yang 2023b (1mg) | mean 1w | 4.6 | 3.5 | 99 | 6.9 | 4.3 | 49 |
| Yang 2023b (1mg) | mean 1w | 4.1 | 4.4 | 99 | 6.9 | 4.3 | 48 |
| Yao 2020 | mean 1w | 7.5 | 2.2 | 153 | 8.2 | 2 | 155 |
| Zhang 2016 | mean 1w | 57.07 | 13.97 | 30 | 71.57 | 30.49 | 30 |
|  |  |  |  |  |  |  |  |
|  |  |  |  |  |  |  |  |
| Study | outcomes | mean | sd | total | mean | sd | total |
| Alipoor 2021 | mean 4w | 10.84 | 3.48 | 67 | 13.09 | 3.79 | 67 |
| Liu 2023 | mean 6w | 5 | 4.6 | 62 | 5.5 | 3.8 | 61 |
| Liu 2021 | mean 6w | 4. 1 | 0.4 | 59 | 6. 1 | 0.9 | 58 |
| Ma 2019 | mean 6w | 4.9 | 4.3 | 327 | 5.55 | 5 | 327 |
| Monks 2022 | mean 6w | 3.81 | 2.06 | 8 | 9.83 | 2.60 | 7 |
| Sun 2023 (0.2mg) | mean 6w | 5.6 | 2.5 | 135 | 6 | 3.2 | 71 |
| Sun 2023 (0.4mg) | mean 6w | 6 | 2.3 | 135 | 6 | 3.2 | 70 |
| Wang 2023a (0.1mg) | mean 6w | 6. 9 | 4 | 28 | 12. 4 | 5.6 | 10 |
| Wang 2023a (0.2mg) | mean 6w | 2. 6 | 2.4 | 30 | 12. 4 | 5.6 | 10 |
| Wang 2023a (0.4mg) | mean 6w | 2. 9 | 2.8 | 29 | 12. 4 | 5.6 | 9 |
| Xu 2017 | mean 6w | 5.6 | 3.9 | 162 | 5.7 | 4.3 | 163 |
| Yang 2023a | mean 6w | 5.1 | 3.9 | 40 | 8.2 | 3.1 | 40 |
| Yang 2023b (1 mg) | mean 6w | 5.0 | 4.4 | 99 | 6.4 | 5.1 | 49 |
| Yang 2023b (2 mg) | mean 6w | 3.8 | 4.3 | 99 | 6.4 | 5.1 | 48 |
|  |  |  |  |  |  |  |  |
|  |  |  |  |  |  |  |  |
|  |  |  |  |  |  |  |  |
|  |  |  |  |  |  |  |  |
|  |  |  |  |  |  |  |  |
| Study | outcomes | events | Total | events | total |  |  |
| Monks 2022 | diplopia | 1 | 8 | 1 | 7 |  |  |
| Shen 2022 | Diplopia | 0 | 102 | 0 | 100 |  |  |
| Wang 2022a (0.1mg) | diplopia | 0 | 38 | 0 | 39 |  |  |
| Wang 2022a (0.2mg) | diplopia | 0 | 40 | 0 | 39 |  |  |
| Wang 2022a (0.4mg) | diplopia | 0 | 39 | 0 | 39 |  |  |
| Xu 2017 | Diplopia | 4 | 162 | 3 | 163 |  |  |
| Wang 2023a (0.1mg) | diplopia | 0 | 28 | 0 | 29 |  |  |
| Wang 2023a (0.2mg) | diplopia | 0 | 30 | 0 | 29 |  |  |
| Wang 2023a (0.4mg) | diplopia | 0 | 29 | 0 | 29 |  |  |
|  |  |  |  |  |  |  |  |
|  |  |  |  |  |  |  |  |
|  |  |  |  |  |  |  |  |
| Study | outcomes | events | Total | events | total |  |  |
| GE Jianlin 2019 (0.2 mg) | dizzness | 1 | 120 | 1 | 60 |  |  |
| GE Jianlin 2019 (0.8mg) | dizzness | 1 | 120 | 0 | 60 |  |  |
| Han 2022 | Dizzy | 15 | 122 | 14 | 153 |  |  |
| Liu 2013 | dizzness | 2 | 40 | 3 | 40 |  |  |
| Liu 2021 | Dizziness | 3 | 59 | 2 | 58 |  |  |
| Liu 2023 | Dizziness | 6 | 62 | 4 | 61 |  |  |
| Luo 2019 | dizzness | 3 | 40 | 2 | 40 |  |  |
| Lv 2015 | dizzness | 2 | 47 | 3 | 47 |  |  |
| Ma 2019 | dizziness | 18 | 327 | 14 | 327 |  |  |
| Monks 2022 | dizziness | 3 | 8 | 1 | 7 |  |  |
| Shen 2022 | dizziness | 34 | 102 | 0 | 100 |  |  |
| Sun 2023 (0.2mg) | Dizziness | 11 | 135 | 7 | 71 |  |  |
| Sun 2023 (0.4mg) | Dizziness | 15 | 135 | 6 | 70 |  |  |
| Wu 2023 | Dizziness | 5 | 120 | 4 | 120 |  |  |
| Xu 2017 | Dizziness | 17 | 162 | 8 | 163 |  |  |
| Yang 2023b (1mg) | Dizziness | 4 | 99 | 2 | 49 |  |  |
| Yang 2023b (2 mg) | Dizziness | 3 | 99 | 1 | 48 |  |  |
| Yao 2020 | dizziness | 2 | 153 | 1 | 155 |  |  |
|  |  |  |  |  |  |  |  |
|  |  |  |  |  |  |  |  |
|  |  |  |  |  |  |  |  |
|  |  |  |  |  |  |  |  |
|  |  |  |  |  |  |  |  |
| Study | outcomes | events | Total | events | total |  |  |
| Liu 2021 | hallucination | 1 | 59 | 0 | 58 |  |  |
| Liu 2023 | Hallucinations | 2 | 62 | 0 | 61 |  |  |
| Ma 2019 | hallucination | 4 | 327 | 0 | 327 |  |  |
| Shen 2022 | Hallucinations | 0 | 102 | 0 | 100 |  |  |
| Sun 2023 (0.2mg) | hallucination | 0 | 135 | 0 | 71 |  |  |
| Sun 2023 (0.4mg) | hallucination | 3 | 135 | 0 | 70 |  |  |
| Xu 2017 | Hallucination | 3 | 162 | 0 | 163 |  |  |
| Yao 2020 | hallucination | 1 | 153 | 0 | 155 |  |  |
|  |  |  |  |  |  |  |  |
|  |  |  |  |  |  |  |  |
| Study | outcomes | events | Total | events | total |  |  |
| Han 2022 | Headache | 5 | 122 | 5 | 153 |  |  |
| Liu 2021 | headache | 1 | 59 | 2 | 58 |  |  |
| Xu 2017 | Headache | 3 | 162 | 0 | 163 |  |  |
| Yao 2020 | headache | 2 | 153 | 1 | 155 |  |  |
|  |  |  |  |  |  |  |  |
|  |  |  |  |  |  |  |  |
| Study | outcomes | events | Total | events | total |  |  |
| GE Jianlin 2019 (0.2 mg) | nausea | 5 | 120 | 2 | 60 |  |  |
| GE Jianlin 2019 (0.8mg) | nausea | 5 | 120 | 2 | 60 |  |  |
| Han 2022 | Nausea | 10 | 122 | 9 | 153 |  |  |
| Liu 2013 | nausea | 1 | 40 | 2 | 40 |  |  |
| Liu 2021 | Nausea | 3 | 59 | 3 | 58 |  |  |
| Liu 2023 | Nausea | 11 | 62 | 15 | 61 |  |  |
| Luo 2019 | nausea | 3 | 40 | 5 | 40 |  |  |
| Lv 2015 | nausea | 1 | 47 | 2 | 47 |  |  |
| Ma 2019 | vomiting | 62 | 327 | 30 | 327 |  |  |
| Monks 2022 | Nausea | 4 | 8 | 2 | 7 |  |  |
| Shen 2022 | Nausea | 7 | 102 | 3 | 100 |  |  |
| Sun 2023 (0.2mg) | Nausea | 28 | 135 | 6 | 71 |  |  |
| Sun 2023 (0.4mg) | Nausea | 35 | 135 | 5 | 70 |  |  |
| Wang 2022 (0.1mg) | nausea | 4 | 38 | 4 | 13 |  |  |
| Wang 2022 (0.2mg) | nausea | 4 | 40 | 4 | 13 |  |  |
| Wang 2022 (0.4mg) | nausea | 3 | 39 | 3 | 13 |  |  |
| Wang 2023a (0.1mg) | nausea | 1 | 28 | 2 | 10 |  |  |
| Wang 2023a (0.2mg) | nausea | 1 | 30 | 2 | 10 |  |  |
| Wang 2023a (0.4mg) | nausea | 1 | 29 | 2 | 9 |  |  |
| Wang 2023b | nausea | 3 | 58 | 4 | 57 |  |  |
| Wu 2023 | Nausea | 11 | 120 | 13 | 120 |  |  |
| Xu 2017 | Vomiting | 1 | 162 | 2 | 163 |  |  |
| Yang 2023b (1mg) | Nausea | 0 | 99 | 2 | 49 |  |  |
| Yang 2023b (2 mg) | Nausea | 1 | 99 | 1 | 48 |  |  |
| Yao 2020 | vomiting | 1 | 153 | 0 | 155 |  |  |
|  |  |  |  |  |  |  |  |
|  |  |  |  |  |  |  |  |
| Study | outcomes | events | Total | events | total |  |  |
| Han 2022 | Drowsiness | 13 | 122 | 10 | 153 |  |  |
| Monks 2022 | sedation | 3 | 8 | 3 | 7 |  |  |
| Wang 2023b | drowsiness | 1 | 58 | 1 | 57 |  |  |
| Xu 2017 | Drowsiness | 5 | 162 | 1 | 163 |  |  |
|  |  |  |  |  |  |  |  |
|  |  |  |  |  |  |  |  |
| Study | outcomes | events | Total | events | total |  |  |
| Liu 2023 | Pruritus | 1 | 62 | 2 | 61 |  |  |
| Luo 2019 | itching | 1 | 40 | 0 | 40 |  |  |
| Monks 2022 | pruritus | 2 | 8 | 2 | 7 |  |  |
| Wang 2022a (0.1mg) | itching | 0 | 38 | 1 | 10 |  |  |
| Wang 2022a (0.2mg) | itching | 0 | 40 | 0 | 10 |  |  |
| Wang 2022a (0.4mg) | itching | 0 | 39 | 0 | 9 |  |  |
